# Juvenile hormone receptor relies on conserved domain interfaces for dimerization-dependent activity

**DOI:** 10.1073/pnas.2605755123

**Published:** 2026-08-06

**Authors:** Lenka Bittova, Sarka Tumova, Mykola Yatsenko, Raveendra Babu Mokhamatam, Roman Tuma, Marek Jindra

**Affiliations:** ^a^Department of Molecular Physiology and Genetics, Institute of Entomology, Biology Center of the Czech Academy of Sciences, Ceske Budejovice 37005, Czech Republic; ^b^Department of Chemistry, Faculty of Science, University of South Bohemia, Ceske Budejovice 37005, Czech Republic; ^c^Preagon Biotech Limited, Zahornice 28903, Czech Republic

**Keywords:** juvenile hormone receptor, bHLH-PAS transcription factor, PAS-A domain, dimerization, *Drosophila*

## Abstract

Juvenile hormone (JH) is vital to insects. In *Drosophila*, JH signals through a basic helix–loop–helix-PER-ARNT-SIM (bHLH-PAS) protein methoprene-tolerant (MET) and its paralog germ cell-expressed (GCE). Binding of JH to the PAS-B domain of MET or GCE triggers the assembly of a DNA-bound, transcriptionally active JH receptor (JHR) heterodimer comprising either MET or GCE and their partner taiman (TAI). Molecular mechanisms of the JHR complex activation remain unclear. Here, we use the *Drosophila* GCE protein to address the presumably critical but as yet unexplored function of the other PAS domain (PAS-A) in JHR signaling. We show that charged amino acid residues, highly conserved within the PAS-A domain across insect and mammalian bHLH-PAS proteins, are required for JHR signaling in vitro and in vivo. Mutations at these sites do not affect the ability of GCE to bind JH to its PAS-B domain. However, they compromise the JH-induced nuclear import of GCE, its dissociation from the chaperone heat-shock protein HSP83, and dimerization with TAI, consequently preventing the DNA binding and transcriptional JHR activities. Functionality of the mutated GCE variants in developing flies is also impaired. Molecular modeling suggests that mutations of the charged GCE PAS-A residues alter the natural salt bridges critical for intra- and interdomain interactions. This in turn causes non-native configurations of both GCE PAS domains, making them incompatible with TAI dimerization and possibly augmenting GCE attachment to HSP83. These results suggest that JHR signaling critically depends on interactions between PAS-A and PAS-B domains which are allosterically coupled.

## Significance

Juvenile hormone (JH) sustains development and reproduction of insects, the largest of animal taxa that bears tremendous impact on ecosystems, agriculture, and transmission of infectious diseases. JH action depends on receptor proteins that, when bound by the hormonal ligand, form a gene-regulatory nuclear complex. Mechanisms of the JH receptor activation remain unclear. Our study identifies evolutionarily conserved sites within the JH receptor protein that are essential for its ligand-dependent transition from a cytoplasmic chaperone assembly to the DNA-bound complex that actively engages in transcriptional regulation. These results provide insights essential for our understanding of molecular principles that govern JH signaling and underlie the effects of the vital endocrine signal.

Juvenile hormones (JHs) are sesquiterpenoids with vital roles in diverse aspects of insect development, reproduction, and physiology (1). In *Drosophila melanogaster*, JH is essential for the completion of metamorphosis to the adult stage (2, 3). JH attains most of its effects through binding to a basic helix-loop-helix–PER-ARNT-SIM (bHLH-PAS) protein called methoprene-tolerant (MET) (1, 4–6). In *Drosophila*, MET arose via gene duplication from *germ cell-expressed* (*gce*), and both GCE and MET are functional in JH signaling (3, 7). Orthologs of MET/GCE occur throughout arthropods but not in vertebrates, and thus far represent the sole example of hormone receptors in the bHLH-PAS protein family (8). To form a transcriptionally active receptor of JH, the ligand-binding MET/GCE monomer dimerizes with another bHLH-PAS protein (1, 6, 8). We will hereafter use the term JH receptor (JHR) to refer to the heterodimeric complex, of which MET/GCE is the essential component.

To carry out their key roles in development and homeostasis, bHLH-PAS proteins form heterodimers capable of binding gene-regulatory DNA elements (8, 9). Besides the basic DNA-binding motif, they possess tandemly arranged PAS domains A and B, both of which engage in dimerization between compatible bHLH-PAS partners and interactions with additional proteins. The PAS-B domain also harbors a cavity capable of binding small-molecular ligands (8, 10–12). The mammalian aryl hydrocarbon receptor (AHR) (13, 14) as well as the insect MET/GCE proteins (6, 7, 15–18) require binding of specific agonists to be activated.

The JH-dependent activation of the JHR resembles the action of AHR which, upon agonist binding, transforms from a cytoplasmic chaperone complex containing the heat-shock protein 90 (HSP90) to a nuclear, DNA-bound heterodimer with its partner ARNT (13, 14). In the absence of JH, MET/GCE rests stabilized in a cytoplasmic complex with HSP83 (an insect ortholog of HSP90), and JH stimulates its nuclear import (19–23). The binding of JH to the PAS-B cavity of MET/GCE induces a conformational change (17, 18) and dissociation from HSP83 (24). Concomitantly, either MET or GCE can form active JHR heterodimers with another bHLH-PAS protein taiman (TAI) (16, 23–26). TAI is an insect ortholog of the vertebrate nuclear receptor coactivators (NCoAs) (8, 27). Both MET/GCE and TAI are required for recognition and DNA binding of JH response elements (JHREs) of JH-inducible genes such as *Krüppel-homolog 1* (*Kr-h1*) (15, 19, 25, 26). Therefore, JH-dependent MET/GCE dimerization with TAI is essential for the JHR transcriptional activity.

Although basic elements of JH signaling have been characterized, critical regulatory mechanisms remain unknown. Notably, the protein–protein interactions of MET/GCE and the role of the PAS-A domain in JHR activation are poorly understood. Judging from rough deletion analyses, the PAS-A domains of both MET and TAI appear to be required (25), but not sufficient (4), for the dimerization. While JHR structure has not been resolved, our molecular modeling aided by AlphaFold-Multimer (28) has predicted a tight association between the PAS domains of GCE and TAI (18). However, the contribution of MET/GCE PAS-A to the JHR complex formation has not been addressed directly.

Crystallography studies on other bHLH-PAS complexes, such as CLOCK:BMAL1 (29) and the heterodimers of ARNT with hypoxia-inducible factor HIF-2α (30), AHR (14, 31, 32) and other bHLH-PAS partners (13, 33) have indicated that the PAS-A domains form inter- and intramolecular interfaces essential for dimerization. Guided by the structural information, the above studies have supported the predicted function of selected amino acid residues through site-directed mutagenesis followed by functional assays. Most of the PAS-A mutations characterized so far have targeted nonpolar residues involved in hydrophobic interactions, although several critical charged residues have also been identified, particularly in the C-terminal β-strands of the PAS-A domains (11, 30, 31, 34).

In this study, we investigated the role of the PAS-A domain in JHR signaling by the *Drosophila* GCE. Considering structures available for mammalian bHLH-PAS proteins and our preliminary structural models, we focused on a C-terminal region of the GCE PAS-A, rich in charged residues that are well conserved across the bHLH-PAS family. We show that some of the pinpointed residues are critical for JHR function in vitro and in vivo. Mutations of these sites compromise the JH-dependent nuclear import of GCE, its dissociation from HSP83, dimerization with TAI, and the JHR transcriptional activity, all without affecting the binding of JH to the PAS-B domain. Our models employing molecular dynamics (MD) simulations suggest plausible mechanisms underlying the deleterious effects of the PAS-A mutations on JHR signaling.

## Results

### Specific Charged Residues Within GCE PAS-A Required for JHR Transcriptional Activity.

**Fig. 1.**
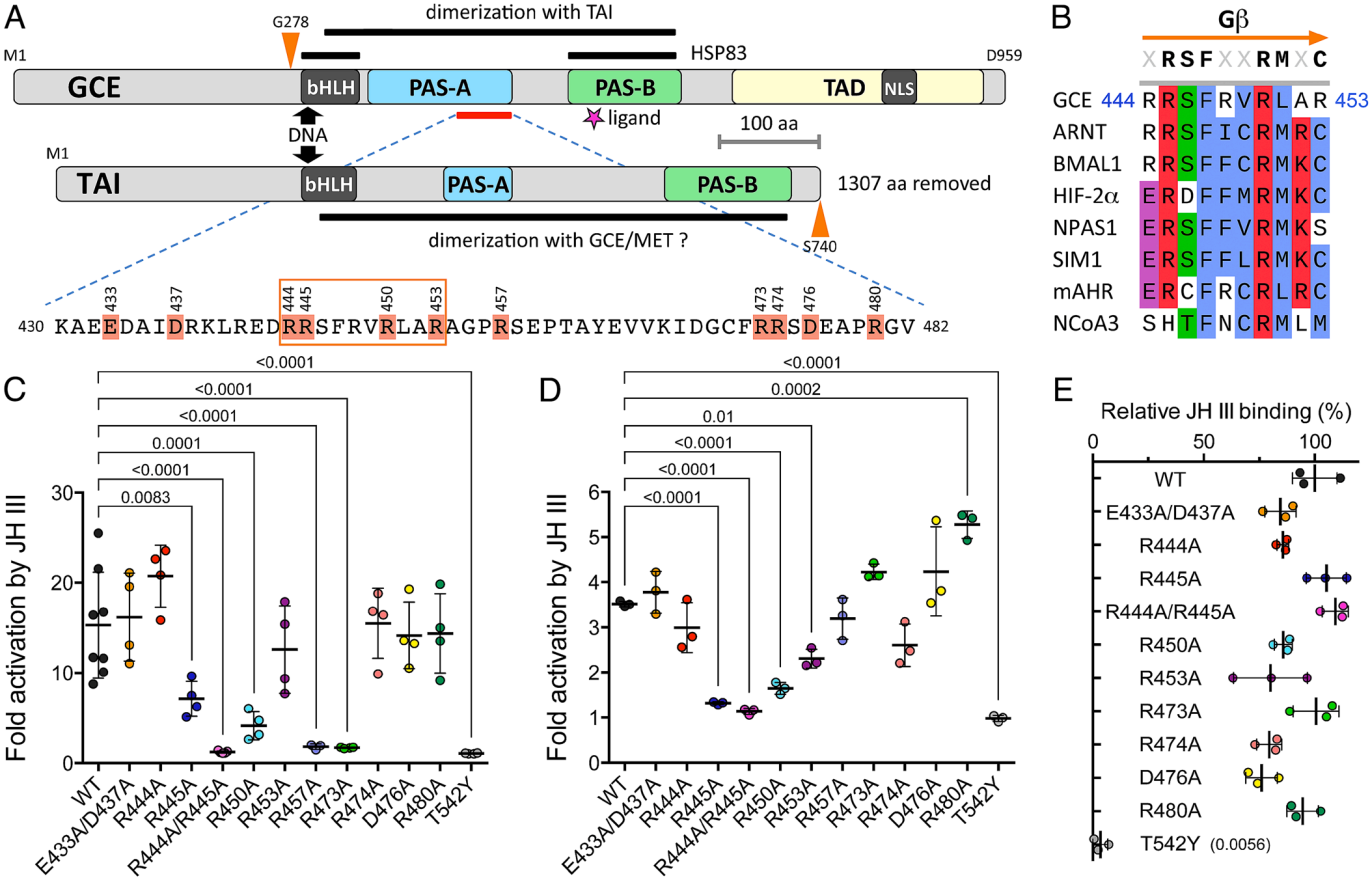
Mutations of conserved arginine residues within the GCE PAS-A domain disrupt JHR function. (*A*) Schematic of the functional domains of the *Drosophila* GCE and TAI proteins with interacting regions roughly delimited by the black bars. The red bar marks the region of the GCE PAS-A subjected to mutagenesis; the residues highlighted in pink were mutated to alanine. The sequence corresponding to the Gβ strand (*SI Appendix*, Fig. S1) is framed. The wild-type (WT) and mutated variants of full-length GCE (M1-D959) were used in the *JHRE-luc* reporter (*C*) and two-hybrid (*D*) assays; JH binding (*E*) was performed with N-terminally truncated GCE (G278-D959). TAI, used in two-hybrid assays (*D*), was truncated after S740 (*Materials and Methods*). The arrowheads indicate the sites of truncation. (*B*) The conserved motif from R444 to R453 in GCE aligned with the Gβ strand (arrow) of the PAS-A fold, previously resolved for some of the indicated mammalian bHLH-PAS proteins, e.g., ARNT, HIF-2α and murine AHR (mAHR) (30, 34). NCoA3 is a human ortholog of TAI. For entire PAS-A alignments, see *SI Appendix*, Fig. S1. (*C* and *D*) *JHRE-luc* transcriptional reporter (*C*) and two-hybrid (*D*) assays were performed with the indicated GCE variants in *Drosophila* Kc167 and in human HEK293 cells, respectively. Activation by JH III (100 nM) is plotted as fold change relative to the luciferase activity measured for each GCE variant in the presence of solvent (ethanol) alone, a value arbitrarily set to 1. (*E*) JH III binding by the in-vitro translated GCE protein variants, plotted relative to the amount of [^3^H]-JH III bound by the control (WT) GCE protein (100%). All data in (*C–E*) appear as mean ± SD from ≥ 4 (*C*) or 3 (*D* and *E*) independent experiments; *P* values (one-way ANOVA) are indicated for significant differences from the control (WT).

The sequence of GCE (*SI Appendix*, Fig. S1) and its structural models (18) are in line with the established structures of bHLH-PAS proteins such as ARNT, HIF-2α and AHR. The PAS-A domain of GCE adopts a conserved fold in which several α-helices pack against a five-stranded antiparallel β-sheet (30, 31, 34). Guided by our preliminary homology modeling and by sequence conservation between *Drosophila* GCE and mammalian bHLH-PAS family members (Fig. 1 *A* and *B* and *SI Appendix*, Fig. S1) and among GCE/MET orthologs across insect evolution (*SI Appendix*, Fig. S2), we focused on the predicted strand Gβ of the GCE PAS-A domain. We mutated to alanine the charged residues within the putative Gβ strand and in its vicinity (Fig. 1*A*).

To gain primary insight into how these mutations might affect JHR signaling, we tested the mutated GCE variants in a JHRE-driven luciferase reporter assay (7, 16) in the *Drosophila* Kc167 cell line. Induction of the *JHRE-luc* reporter by JH requires the endogenous *Drosophila* GCE, TAI, and HSP83 proteins, as RNAi-mediated knockdown of either one prevented JH from inducing *JHRE-luc* (*SI Appendix*, Fig. S3). Consistent with a low level of *Met* mRNA (relative to *gce* mRNA) in these *Drosophila* cells (35), the GCE paralog MET was dispensable for activation of *JHRE-luc* by JH. Therefore, upon knockdown of endogenous GCE, transfected GCE variants could be assessed in the Kc167 cells without appreciable activity of the endogenous MET and GCE proteins.

The GCE PAS-A mutations affected the *JHRE-luc* reporter activation by JH III to various extent (Fig. 1*C*). Individual mutations of four arginine residues (R445, R450, R457, R473) to alanine severely impaired GCE function. An interesting synergy occurred with the R444A mutation, alone without a negative effect, which enhanced the impact of R445A, rendering the R444A/R445A double-mutant receptor inactive in the assay (Fig. 1*C*). The previously described PAS-B mutation T542Y that prevents GCE from binding to JH III (7) was equally inactive. The remaining PAS-A mutations had no statistically significant effect. These data indicate that some of the conserved arginine residues are important for the transcriptional activity of the JHR.

### Mutations of the GCE PAS-A Charged Residues Prevent Interaction with TAI.

Transcriptional function of the JHR complex requires dimerization of the GCE and TAI monomers. Therefore, one explanation for the observed loss of activity of the GCE PAS-A mutated variants might be their failure to dimerize with TAI. To test whether the mutations affect JHR complex assembly, we employed an established agonist-dependent two-hybrid assay (16, 36). In this assay, the *Drosophila* JHR proteins fused with the GAL4 DNA-binding (TAI) and VP16 activation (GCE) domains were coexpressed together with a UAS-driven luciferase reporter in the human HEK293 cell line.

Consistent with the *JHRE-luc* reporter data, the R445A, R450A, and R444A/R445A variants were unable to effectively dimerize with TAI in the presence of JH III (Fig. 1*D*), suggesting that dimerization was required for JHR transcriptional activity. The R444A/R445A double mutation again had the strongest effect, comparable to the GCE^T542Y^ variant deficient in JH III binding. Importantly, all mutations that were tolerated in the *JHRE-luc* assay also permitted GCE:TAI dimerization, except for the partial but significant effect of R453A (cf. Fig. 1 *C* and *D*). Conversely, however, the R457A and R473A variants failed to induce *JHRE-luc* even though they interacted with TAI, indicating that their transcriptional activity may be disrupted for reasons other than GCE:TAI dimerization. Together, the data demonstrate that the conserved R445 and R450 residues in the putative Gβ strand of the GCE PAS-A are indispensable for a productive JHR assembly and signaling.

### JH Binding by the GCE PAS-B Domain Is Unaffected by the PAS-A Mutations.

The JH-binding capacity of GCE is necessary for transcriptional activation and to stimulate and stabilize dimerization with TAI (16). Although the PAS-A domain is not essential for JH binding (4, 7) mutations in it could lead to improper folding and instability of the entire GCE protein. It was therefore important to determine whether the PAS-A mutations that impair the JH-dependent *JHRE-luc* induction and/or the JHR complex assembly retain the JH binding function.

Using an established in vitro binding assay with radiolabeled [^3^H]-JH III (4, 37), we compared JH-binding activity of the PAS-A GCE mutants with those of the control GCE^WT^ and the ligand-nonbinding GCE^T542Y^ proteins. Unlike T542Y, none of the mutations blocked the GCE capacity to bind JH III (Fig. 1*E*), indicating that the PAS-A variants retained a degree of structural integrity sufficient for this PAS-B domain function. The data also suggest that the ligand binding function is independent of JHR dimerization and signaling, which are selectively disrupted by the PAS-A mutations.

### Mutations of Specific GCE PAS-A Residues Impact *Drosophila* Development.

The JHR proteins MET/GCE are required for *Drosophila* to complete metamorphosis, as the *Met gce* double-mutant flies (combining the alleles *Met^27^* and *gce^2.5k^*) all die at the pupal stage (3, 7). Normal development of the *Met^27^ gce^2.5k^* double mutants can be restored by transgenic expression of a functional variant of either MET or GCE, driven by the *GAL4-UAS* system under a ubiquitous but moderate-strength *armadillo* (*arm*) enhancer (7). We have used this genetic rescue to assess in vivo functionality of selected GCE PAS-A variants, focusing on those mutations that disrupt JHR signaling (R445A, R444A/R445A, R450A, R473A) while taking GCE^E433A/D437A^ as a theoretically neutral mutation and GCE^T542Y^ as the inactive control.

**Fig. 2.**
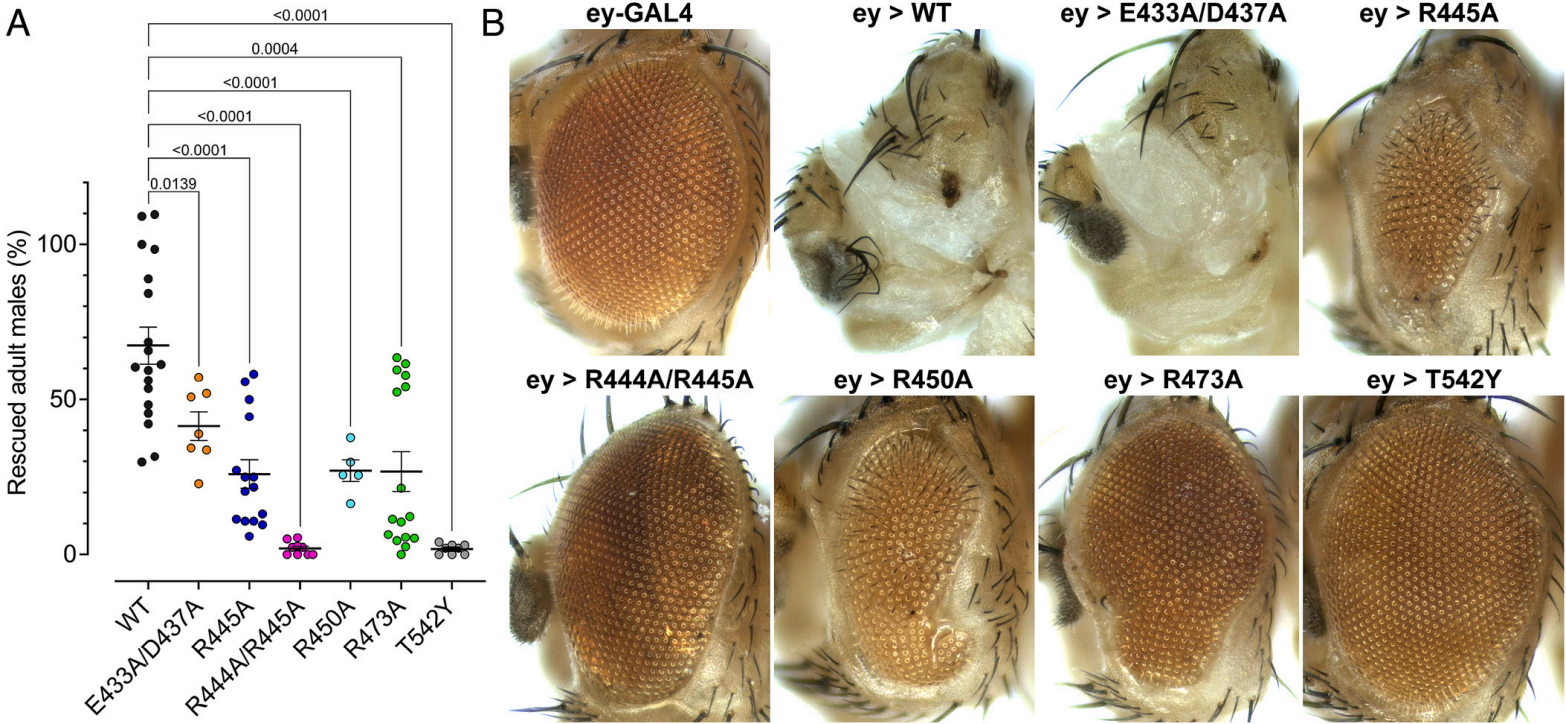
Validation of selected GCE variants in vivo. (*A*) Genetic rescue of *Met^27^ gce^2.5k^/Y* mutant males by ubiquitous expression of transgenic full-length GCE proteins in the double-mutant background. Neither of the inactive R444A/R445A and T542A variants rescued the mutants; the few emerging escapers resulted from ~2% recombination rate between the *Met^27^ gce^2.5k^* chromosome and the *FM7c* balancer, causing loss of the dominant scoring marker Bar. Each data point represents results from an individual crossing, each with 78 scored males on average. The total number of male progenies scored per genotype was 912 on average. The data are presented as mean ± SEM; *P* values (Dunnett’s multiple comparisons) are indicated. (*B*) Gain-of-function effects on eye development. The full-length GCE variants were ectopically expressed using the *eyeless* driver (*ey-Gal4*) in eye imaginal discs. While GCE^R444A/R445A^ and GCE^T542Y^ caused no appreciable defects, expression of GCE^WT^ or the active E433A/D437A variant led to eyeless flies, of which most were unable to emerge. Intermediate defects caused by the other three mutations corresponded with their partial functionality, and the expressivity of the phenotypes varied (the most representative appearances are shown). About 640 animals were scored per genotype, and the penetrance of the anomalies was total.

All *gce* transgenic constructs were inserted into the same position on the third chromosome. Transgenic males were crossed with balanced *Met^27^ gce^2.5k^/FM7c* females (both *Met* and *gce* reside on the X chromosome), and viable mutant *Met^27^ gce^2.5k^/Y* adult male progeny were scored against their *FM7c/Y* siblings. Equal number of males of both genotypes would be considered a 100% rescue. Fig. 2*A* shows that the GCE PAS-A mutations R445A, R450A, and R473A, which compromised the dimerization or the transcriptional functions of JHR (Fig. 1 *C* and *D*), rescued adult development of the *Met^27^ gce^2.5k^/Y* mutant males to a lesser extent (~27%) relative to ~67% rescue obtained with the GCE^WT^ transgene. The GCE^E433A/D437A^ variant provided an intermediate level (~41%) of genetic rescue. Strikingly, the R444A/R445A double-mutant GCE failed to substitute for the absent MET and GCE proteins in vivo altogether. Like the JH binding deficient GCE^T542Y^ variant, GCE^R444A/R445A^ allowed almost no *Met^27^ gce^2.5k^/Y* adult males to emerge (Fig. 2*A*).

By using additional *GAL4* drivers, we found that in contrast to the rescuing effect of mild ubiquitous expression described above, strong ectopic expression of functional GCE leads to lethality or to visible developmental defects. For instance, misexpression of GCE^WT^, but not the inactive GCE^T542Y^ variant, in the compound eye primordia (eye imaginal discs) under the *eyeless* (*ey-GAL4*) driver compromised eye development (Fig. 2*B*). This gain-of-function phenotype likely results from the fact that GCE expression in the eye imaginal disc is normally very low or undetectable (38) (*SI Appendix*, Fig. S4).

We exploited this gain-of-function effect as another test of functionality of the GCE PAS-A mutants in vivo. The *ey-GAL4* driven expression of either GCE^WT^ or GCE^E433A/D437A^, both active in the cell-based assays (Fig. 1 *C* and *D*), resulted in pharate adult lethality (>99%) with only few eyeless flies emerging (Fig. 2*B*). The single mutations R445A, R450A, and R473A, which significantly compromised JHR function, affected eye development to a milder and variable extent, with R473A being least severe. Finally, misexpression of the GCE^R444A/R445A^ variant that proved inactive in both cell-based assays (Fig. 1 *C* and *D*) and incapable of rescuing JHR-deficient flies (Fig. 2*A*) caused no appreciable eye malformation, similar to GCE^T542Y^ (Fig. 2*B*). Together, these data demonstrate loss of in vivo activity of GCE^R444A/R445A^ and partial loss of function caused by the other GCE PAS-A mutations in a good correlation with the transcriptional and dimerization activities of those GCE variants.

### Nuclear Localization of GCE Is Impaired by Specific PAS-A Mutations.

According to the current model of JHR signaling mechanism (1, 6), a JH-bound form of MET/GCE is imported to the nucleus where it forms a transcriptionally competent heterodimer with TAI. To examine whether selected PAS-A mutations alter cellular localization of the GCE protein in vivo, we stained the salivary glands of *Drosophila* larvae with an anti-GCE antibody. While this antibody revealed nuclear localization of the endogenous GCE in control larvae (*SI Appendix*, Fig. S4), no signal was detected in *gce^2.5k^* null mutant larvae (*SI Appendix*, Figs. S4 and S5).

To visualize the localization of the mutated GCE proteins, we performed the anti-GCE staining in larvae expressing the transgenic GCE variants under a *ptc-GAL4* driver in the *gce^2.5k^* background (*SI Appendix*, Fig. S5). As expected, the transgenic GCE^WT^ protein produced a strictly nuclear signal, and a similar result was obtained with the functional GCE^E433A/D437A^ variant. Appreciable cytosolic staining was detected for GCE mutated at the critical R445 residue, and particularly so when combined with the R444A mutation. A strong cytosolic signal was also noted for the GCE^T542Y^ variant incapable of binding JH (*SI Appendix*, Fig. S5). Surprisingly, GCE^R450A^ showed little cytoplasmic presence. However, the nucleo-cytoplasmic distribution of the mutated proteins varied considerably among individual larvae and salivary gland cells, making a quantitative analysis difficult. Moreover, the endogenous JH titer in larvae could not be controlled.

**Fig. 3.**
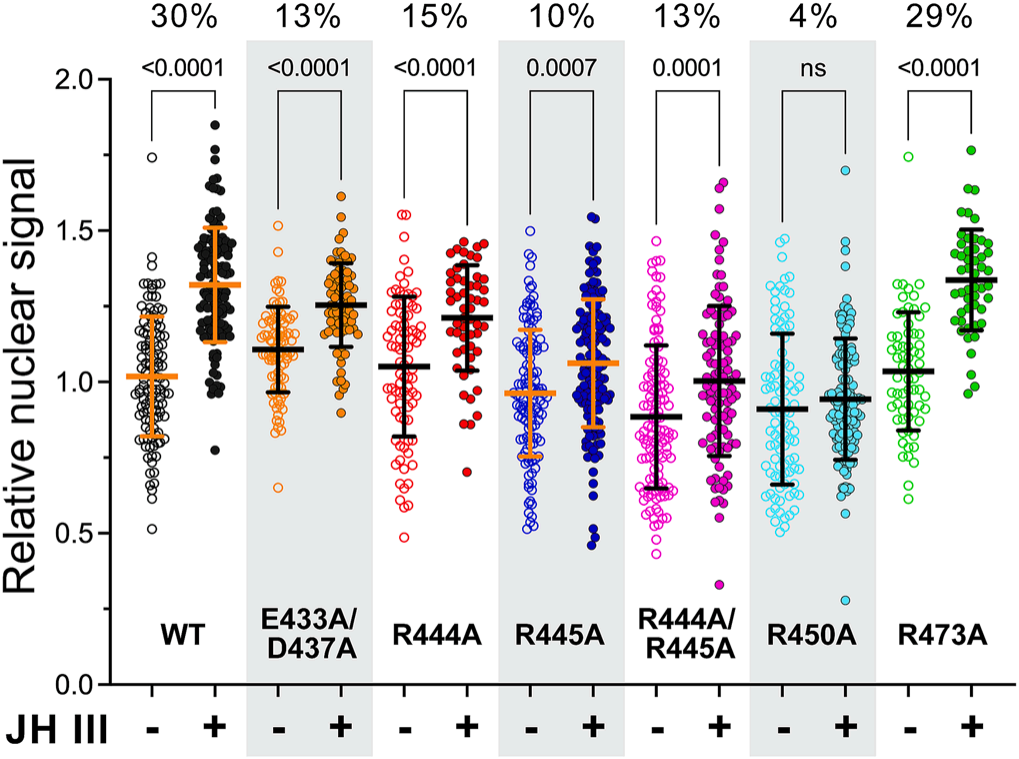
The effect of JH on nuclear localization of WT and mutated full-length GCE proteins. The GCE variants were transiently expressed in HEK293 cells, exposed either to 1 µM JH III (+) or ethanol (−) for 1 h. After immunostaining (*SI Appendix*, Fig. S6), cellular localization of GCE was assessed as integrated pixel densities in confocal slices from representative areas of cells. Each dot represents the nuclear-to-total signal ratio for an individual cell; the average per cent increase of the relative nuclear signal is shown above the columns. All GCE variants except for R450A displayed statistically significant nuclear accumulation in response to JH III, which was markedly reduced relative to GCE^WT^ and GCE^R473A^. The data are presented as mean ± SD. The statistical significance was determined by One-way ANOVA and Šídák’s multiple comparisons test.

To assess cellular localization of mutated GCE proteins free from the influence of endogenous JH, we expressed selected GCE variants, tagged with the Myc epitope, in the human HEK293 cells. Our previous study (24) suggests that in mammalian cells, HSP90 substitutes for HSP83 in stabilizing GCE. Representative images of Myc-GCE localization in the absence and presence of JH III are shown, and GCE distribution is quantified in *SI Appendix*, Fig. S6. As expected, the experiment revealed a strong JH-dependent shift toward nuclear localization for GCE^WT^, which was also significant, albeit at a reduced rate, for the other GCE variants except for R450A (Fig. 3). Interestingly, nuclear localization of GCE was not prevented by the R473A mutation, which abolished transcriptional activity but not GCE:TAI dimerization (Fig. 1 *C* and *D*). The R445A, R450A, and R444A/R445A variants were all notably retained in the cytoplasm of HEK293 cells even upon JH III treatment (*SI Appendix*, Fig. S6). Nuclear translocation of GCE was most markedly impaired by mutating the critical R445 and R450 residues. With the exception of GCE^R450^, the data agree with the GCE antibody staining in the salivary glands (*SI Appendix*, Fig. S5). Given that the PAS-A mutations do not preclude binding of JH III (Fig. 1*E*), the compromised nuclear import of the GCE mutant proteins is independent of the JH-binding capacity.

### PAS-A Mutations Prevent JH-Dependent Dissociation of GCE from HSP83.

Agonist-induced dissociation of the presignaling MET/GCE chaperone complex containing HSP83 appears to be a critical step in the hormonal activation of JHR (1, 6). Indeed, a MET:HSP83 complex from the beetle *Tribolium castaneum* has been shown to dissociate upon JH III addition in a split luciferase assay (NanoBiT) that monitors protein–protein interactions in live cells (24). Failure to undergo this agonist-dependent dissociation could disrupt the formation of an active JHR complex of MET/GCE with TAI.

**Fig. 4.**
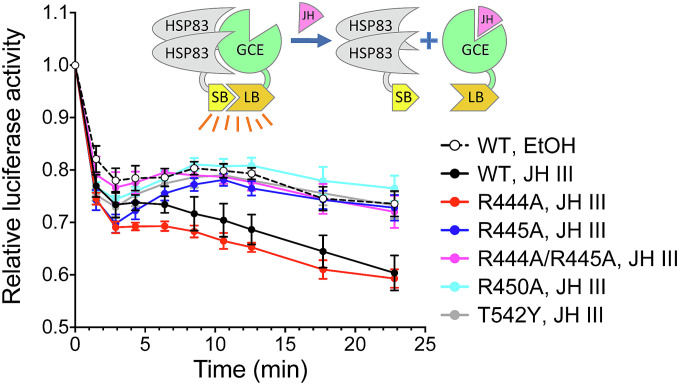
The effects of GCE mutations on JH-triggered dissociation from HSP83 using the NanoBiT assay. The N-terminally truncated GCE (G278-D959; Fig. 1*A*) variants were tagged with NanoLuc luciferase large bit fragment (LB), so when in complex with HSP83, tagged with the NanoLuc small bit (SB), the two tags reconstituted into the active luciferase. Dissociation of the WT and R444A variants from HSP83 upon JH III application occurred over time, measured as a loss of luminescence compared to the vehicle (ethanol) control. In contrast, the R445A, R444A/R445A, R450A, and T542Y mutants failed to dissociate from the chaperone complex when 1 µM JH III was added. The traces are shown as average of three (R444A/R445A) or five independent experiments with the error bars representing SEM. The vehicle baselines for different GCE variants were almost identical; only the trace for the WT GCE vehicle treatment is shown for clarity.

We employed the NanoBiT technique to investigate JH-dependent dissociation of the WT and selected GCE mutant variants from HSP83. When tagged with the small and large fragments of the NanoLuc luciferase, respectively, and coexpressed in the mammalian Chinese hamster ovary (CHO) cell line, HSP83 and all tested GCE variants produced basal levels of luminescence indicative of a mutual interaction. Dissociation of GCE^WT^ from HSP83 became apparent by about 7 min after JH III addition and continued over time, as indicated by a gradual decrease of luminescence relative to the vehicle control (Fig. 4). The same was true for the active variant GCE^R444A^. Strikingly, the PAS-A mutations R445A, R450A, and R444A/R445A all prevented the JH-dependent dissociation of GCE from HSP83 (Fig. 4). As expected, also the GCE^T542Y^ variant failed to effectively dissociate from HSP83 in the presence of JH III, confirming our previous finding that activation of a presignaling JHR chaperone complex requires agonist binding to the PAS-B domain (24).

The NanoBiT assay data suggest that mutation of the specific PAS-A arginine residues alter the structure of GCE in a way that prevents its JH-dependent dissociation from HSP83 and the concomitant nuclear import and dimerization with TAI, both requisite to forming a transcriptionally competent JHR complex.

### GCE^R444A/R445A^ Fails to Form a Functional, DNA-Binding Competent JHR Complex.

Next, we focused on the GCE PAS-A variant bearing the double mutation R444A/R445A, which had the strongest impact on GCE function in the *JHRE-luc* transcriptional assay (Fig. 1*C*) and in vivo (Fig. 2). To validate the notion that the double mutation prevented GCE:TAI dimerization (Fig. 1*D*), we performed coimmunoprecipitation (co-IP) with tagged TAI (EGFP-TAI) and Myc-3xFlag-GCE proteins in HEK293 cell lysates.

**Fig. 5.**
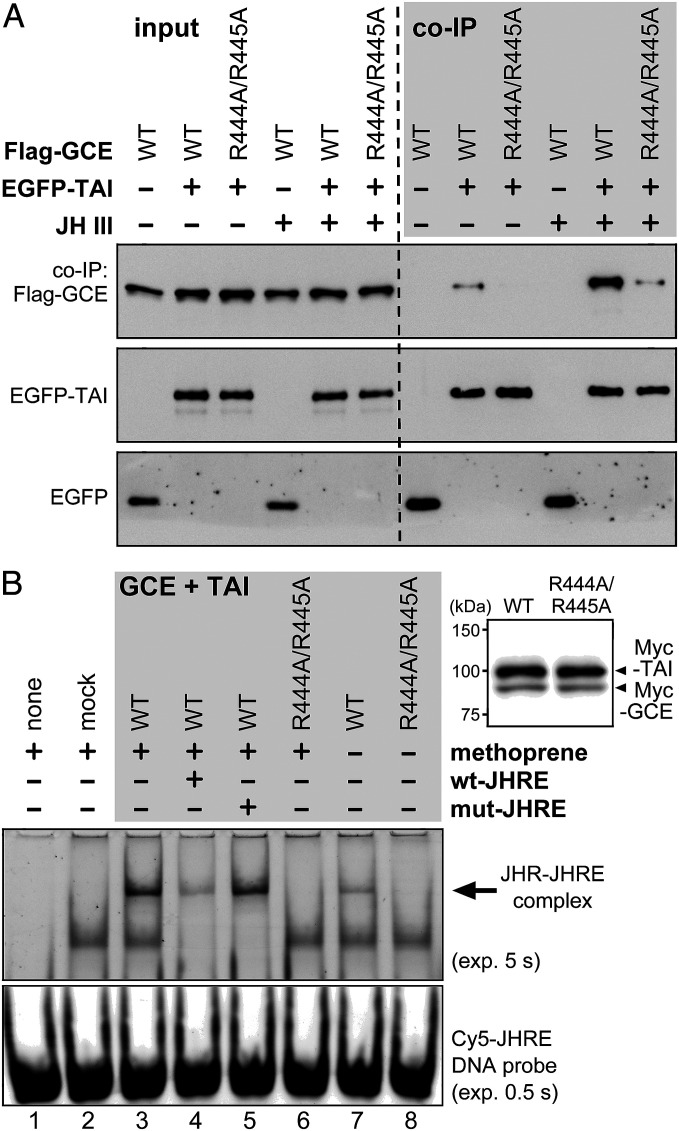
(*A*) JH-induced interaction between TAI and GCE analyzed by co-IP. Either 1 µM JH III or ethanol was added to HEK293 cells coexpressing EGFP-TAI (or EGFP alone) with the Flag-GCE WT or R444A/R445A variants 1 h prior to cell lysis. The complexes were captured using GFP-Trap beads and the interacting proteins detected by immunoblotting with Flag or EGFP antibodies. The input panel represents 1.6% of the starting lysate; 16% of immunoprecipitated material was loaded. The co-IP panel shows low basal interaction between TAI and GCE^WT^ that increased greatly in the presence of JH III, whereas the association between TAI and GCE^R444A/R445A^ was diminished in both vehicle- and JH-treated cells. (*B*) Effect of GCE mutation on the ability to bind JHRE DNA in electrophoretic mobility shift assay (EMSA). TAI was in-vitro translated with WT or R444A/R445A GCE, as verified by immunoblotting (*Top* *Right*). The lysates were incubated with the Cy5-labeled wt-JHRE oligonucleotide. The top part of the gel shows the retarded GCE:TAI:JHRE complex (arrow) formed in the absence or presence of 8.5 µM methoprene. The amount of the detected complex was reduced by adding 10-fold excess of unlabeled wt-JHRE (lane 4) but not the mutated JHRE oligonucleotide (lane 5). Reduced DNA binding was detected in the absence of methoprene (lane 7). The GCE^R444A/R445A^ mutant was unable to form the JHRE-bound complex (lanes 6 and 8). Lanes 1 and 2 contained no protein input and mock-translation lysate, respectively. The bottom part of the same gel shows the unreacted Cy5-JHRE DNA probe, detected with shorter exposure.

Experiments with EGFP-TAI immobilized on GFP-Trap magnetic particles revealed a low level of JH-independent interaction of GCE^WT^ with TAI, which was strongly stimulated by JH III addition (Fig. 5*A*). This result matched previous data on the interaction of MET and TAI from *Tribolium* (4, 23). In contrast, the GCE^R444A/R445A^ variant did not immunoprecipitate with TAI in the absence of JH III, and addition of the hormone led to a low level of association comparable to that observed for GCE^WT^ without JH III (Fig. 5*A*). In a reciprocal setting with Myc-3xFlag-GCE bound to anti-Flag magnetic beads, no immunoprecipitation of TAI was detected for the GCE^R444A/R445A^ mutant even in the presence of JH III, whereas GCE^WT^ interacted with TAI as expected (*SI Appendix*, Fig. S7). Interestingly, the R444A/R445A mutation did not preclude formation of a GCE:GCE complex, which was partially diminished in the presence of JH III (*SI Appendix*, Fig. S8), again indicating proper folding of the mutant protein.

Since MET:TAI dimerization is essential for recognition and binding of specific JHRE DNA (15), we tested whether the R444A/R445A mutation that hinders the GCE:TAI heterodimer assembly also prevents it from binding DNA. EMSA was performed with in vitro translated GCE and TAI proteins, and a 30-bp JHRE DNA (*k-JHRE*) from the JH-inducible *Tribolium* gene *Kr-h1* (39).

First, we sought to verify that neither GCE nor TAI can bind to the JHRE DNA alone. Indeed, the EMSA resolved a slowly migrating complex of the labeled *k-JHRE* probe only when both GCE and TAI proteins were present (*SI Appendix*, Fig. S9). The binding was specific, as the complex was reduced by adding excess unlabeled *k-JHRE* DNA but not its mutated version (Fig. 5*B* and *SI Appendix*, Fig. S9*A*). Consistent with data on the *Tribolium* MET:TAI dimer (23), the DNA binding occurred even in the absence of the JHR agonist methoprene but was enhanced by methoprene addition (Fig. 5*B*), likely through stimulating the association between GCE and TAI. The R444A/R445A mutation of GCE abolished the DNA-binding competence of the JHR complex although both GCE^WT^ and GCE^R444A/R445A^ proteins were expressed to a similar extent (Fig. 5*B* and *SI Appendix*, Fig. S9*B*). These results confirm that the loss of GCE:TAI dimerization blocks the assembly of the DNA-binding, transcriptionally active JHR complex.

### MD Simulation of the Impact of PAS-A Mutations on the GCE:TAI Heterodimer.

**Fig. 6.**
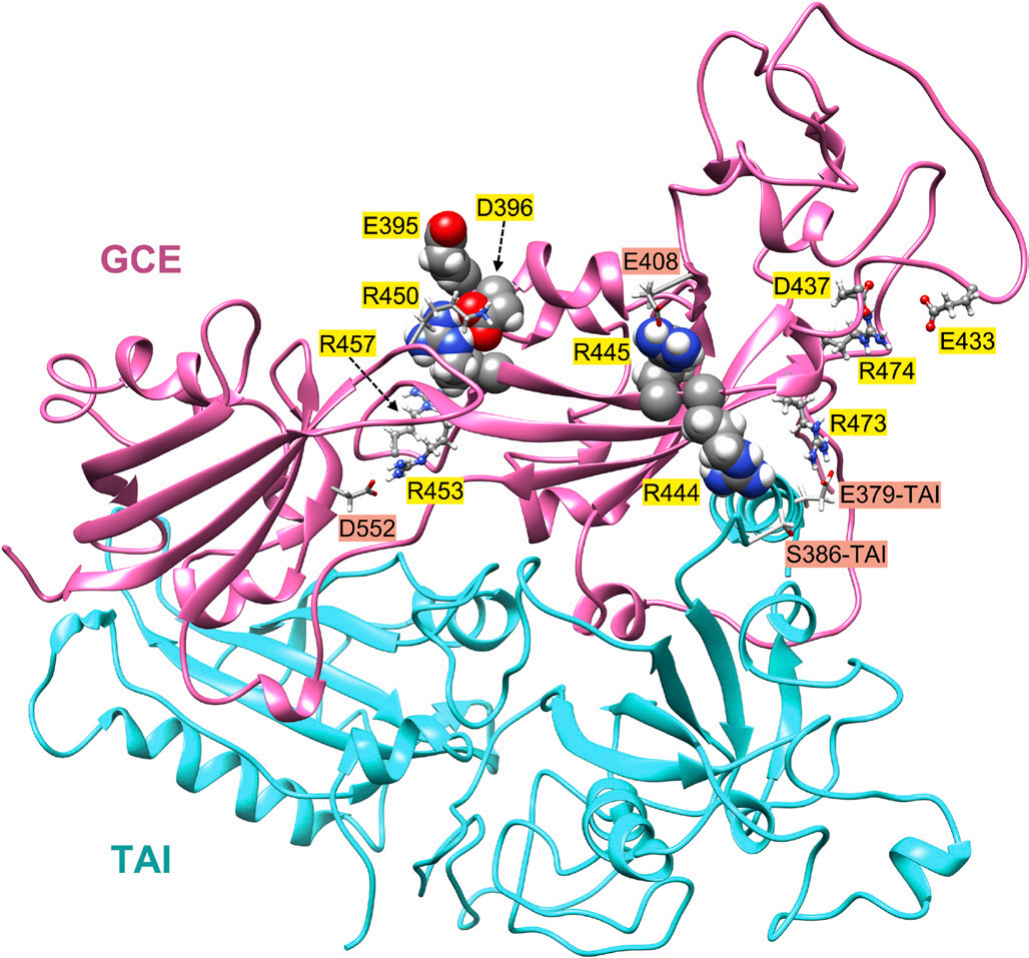
A relaxed model (3-µs all-atom MD simulation) of the structured core of the PAS-A and PAS-B domains of the GCE (pink):TAI (cyan) heterodimer. Selected residues that were mutated in this study are highlighted in yellow, and those highlighted in orange were predicted to interact but were not mutated. Residues whose replacement with alanine causes strong signaling defects are shown in spacefill representation.

To map the mutated PAS-A residues onto the structural framework of the JHR complex, we generated a DNA-bound model of GCE:TAI from the smallest active fragment using AlphaFold 3, further adjusted by core relaxation (*SI Appendix*, Fig. S10). Only the structured, MD-relaxed PAS-A/B fragment of the GCE:TAI complex, called the “core” here, was considered in further analyses as it included all residues of interest and the relevant domain and subunit interfaces. Within the model, the conserved R444 in GCE PAS-A interacts with S386 (located in a helix between the bHLH and PAS-A domains of TAI) or with E442, whereas R445 forms a hydrogen bond with T407 and an intra-domain salt bridge with E408 or D443 (Fig. 6 and *SI Appendix*, Fig. S11). MD simulations in the presence and absence of JH III (*SI Appendix*, Fig. S12) suggest that this charge cluster is not directly affected by ligand binding. However, interaction energy of ligand binding is expected to stabilize the native conformation of the GCE PAS-B domain and, presumably, compete with HSP83 binding which might occlude this domain interface.

To test whether the positive charge of the conserved residues is indeed required for GCE function, we prepared a variant in which both R444 and R445 were mutated to glutamine. Like the arginine-to-alanine double mutant protein (Fig. 1 *C*–*E*), GCE^R444Q/R445Q^ bound JH III but could neither activate *JHRE-luc* nor dimerize with TAI (*SI Appendix*, Fig. S13 *A–C*). Dissociation of the GCE^R444Q/R445Q^ protein from HSP83 was also impaired (*SI Appendix*, Fig. S13*D*).

**Fig. 7.**
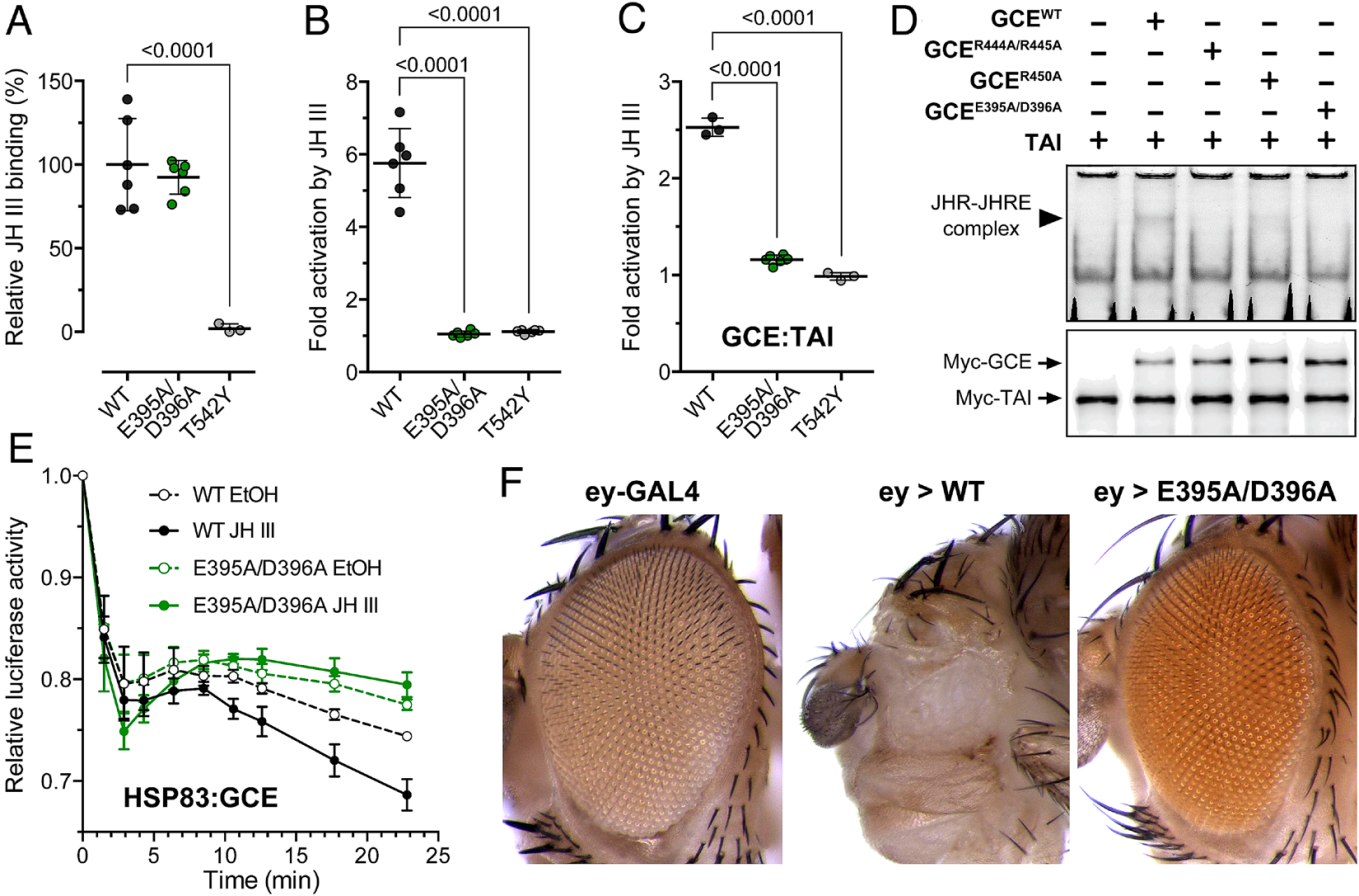
Conserved acidic residues with a predicted stabilizing function in the GCE PAS-A domain are essential for JHR activity. (*A*) Binding of [^3^H]-JH III by the in-vitro translated GCE protein was unaffected by substituting E395 and D396 with alanine relative to the control (WT) GCE (100%); the GCE^T542Y^ mutant is a JH nonbinding control. (*B*) GCE^E395A/D396A^ was unable to induce the *JHRE-luc* reporter in response to JH III (100 nM) in the Kc167 cells. Data are plotted relative to the luciferase activity with vehicle alone. (*C*) GCE^E395A/D396A^ failed to dimerize with TAI in the two-hybrid assay. Activation by JH III (100 nM) is plotted as fold change relative to the luciferase activity measured for each GCE variant in the presence of vehicle alone, a value arbitrarily set to 1. (*D*) Like R444A/R445A (cf. Fig. 5*B*), the E395A/D396A mutation prevented GCE and TAI from binding to the JHRE in EMSA; the level of binding also appeared to be reduced by the R450A mutation. Note that TAI alone did not interact with the DNA. Immunoblot (*Bottom*) shows the in-vitro translated proteins used in the assays. Methoprene (8.5 µM) was present in all reactions. (*E*) JH-induced dissociation of GCE from HSP83 was impaired by the E395A/D396A mutation relative to GCE^WT^ as determined in the NanoBiT assay in the presence or absence of 1 µM JH III. The traces are shown as average of four independent experiments with the error bars representing SEM. (*F*) While the ectopic expression of GCE^WT^ resulted in predominant lethality with few emerging eyeless adults, the GCE^E395A/D396A^ variant caused no such defects, similar to the *ey-Gal4* driver control alone. Data in (*A–C* and *E*) appear as mean ± SD from numbers of independent measurements indicated by the dots; *P* values (one-way ANOVA) are shown only for significant differences.

The invariably conserved R450 residue is predicted to form intra-molecular charge clusters with K630 (PAS-B) and with E395/D396 within the putative Fα helix of GCE PAS-A (Fig. 6). Of these residues, E395 is either conserved or substituted by aspartic acid in most insect orthologs; D396 is totally conserved across insect and human bHLH-PAS proteins (*SI Appendix*, Figs. S1 and S2). We hypothesized that mutating the conserved E395 and D396 should block JHR signaling. Indeed, while capable of binding JH III (Fig. 7*A*), the GCE^E395A/D396A^ variant failed to induce JH-dependent transcription, form a DNA-binding dimer with TAI, and dissociate from HSP83 in functional assays (Fig. 7 *B*–*E*). It also tended to be retained in the cytoplasm when compared to GCE^WT^ (*SI Appendix*, Fig. S14). Unlike GCE^WT^, the E395A/D396A mutant protein did not disrupt development of the compound eye when overexpressed in transgenic flies, indicating that it lacked functionality in vivo (Fig. 7*F*). Mutating E395 and D396 had effects similar to alanine substitution of the conserved R450 (Figs. 1 *C* and *D* and 4), which is predicted to interact with E395 and D396 (Fig. 6). Thus, the computational GCE:TAI dimer model alluded to the critical role for some of the charged residues.

Since the conserved arginines as well as the E395 and D396 reside close to the PAS-A/B domain interfaces (Fig. 6), their mutations could destabilize the GCE fold and consequently affect interactions with TAI or HSP83. We tested the effect of the mutations within the framework of AlphaFold 3 core models by extensive microsecond MD simulations after introducing mutations in silico; control (WT) models were subjected to the same protocol. The results are detailed in *SI Appendix*, Figs. S11, S15, and S16. Briefly, the electrostatic network of salt bridges stabilizing the WT GCE:TAI heterodimer (*SI Appendix*, Fig. S11 *A–D*) was disrupted by the R444A/R445A mutation, leading to alternative domain configurations (*SI Appendix*, Fig. S11 *E–G*). The PAS-A hydrophobic core (F447, F472) was exposed and non-native interactions formed between E442/D443 and positively charged residues in adjacent parts of the GCE PAS-A or B domains. These changes were accompanied by repositioning of the TAI PAS-B domain (*SI Appendix*, Fig. S11*A*).

The structural consequences of the E395A/D396A mutation were explored using a reduced model encompassing PAS-A and B domains of GCE alone. The E395A/D396A mutant noticeably departed from the WT configuration, adopting an altered domain disposition with at least two main steady state configurations, showing different PAS-B domain positions and interfacial interactions (*SI Appendix*, Fig. S15). Mutating R450 led to a similar PAS-B displacement and interface remodeling (*SI Appendix*, Fig. S16 *A–C*). The departure from the average WT interface configuration was further illustrated by distance distributions between the PAS-A Fα helix with the conserved E395/D396 and a PAS-B loop containing K630 (*SI Appendix*, Fig. S16*D*). The distributions in both the E395A/D396A and R450A mutant models were bimodal, reflecting the alternative stationary structures relative to the WT protein. In summary, our models suggest that the mutationally induced structural changes likely disrupt local domain and dimer interfaces and may destabilize the JHR assembly.

## Discussion

Activation of bHLH-PAS transcription factors relies on their functional domains interacting with multiple proteins and DNA. The mammalian AHR and insect JHR additionally depend on binding of small-molecular agonists which trigger a sequence of events transforming either receptor from a cytoplasmic, chaperone associated monomer to the DNA binding dimer with the cognate bHLH-PAS partner (6, 13, 14). For the JHR, amino acid residues in the PAS-B domain of MET/GCE have been identified as critical for JH binding; mutating these residues (such as T542 in GCE) impairs the agonist-dependent JHR signaling activities (4, 7, 15, 18, 24, this study). Directed mutagenesis of the MET and TAI N-terminal basic regions has further shown that specific amino acids in either of the interacting partners are required to recognize and bind the JHRE DNA (15).

Relative to the ligand- and DNA-binding functions, protein–protein interactions of the JHR proteins remain poorly studied. Based on structural data available on heterodimers of ARNT with AHR (14, 31, 32) and other bHLH-PAS family members (11, 30, 33), the PAS-A domains essentially engage in dimerization of the partner pairs. To address the previously uncharacterized role of the PAS-A domain in JHR signaling, we subjected *Drosophila* GCE PAS-A to mutagenesis of selected conserved residues on the presumed protein interfaces and tested the variants for activity in several functional assays.

**Fig. 8.**
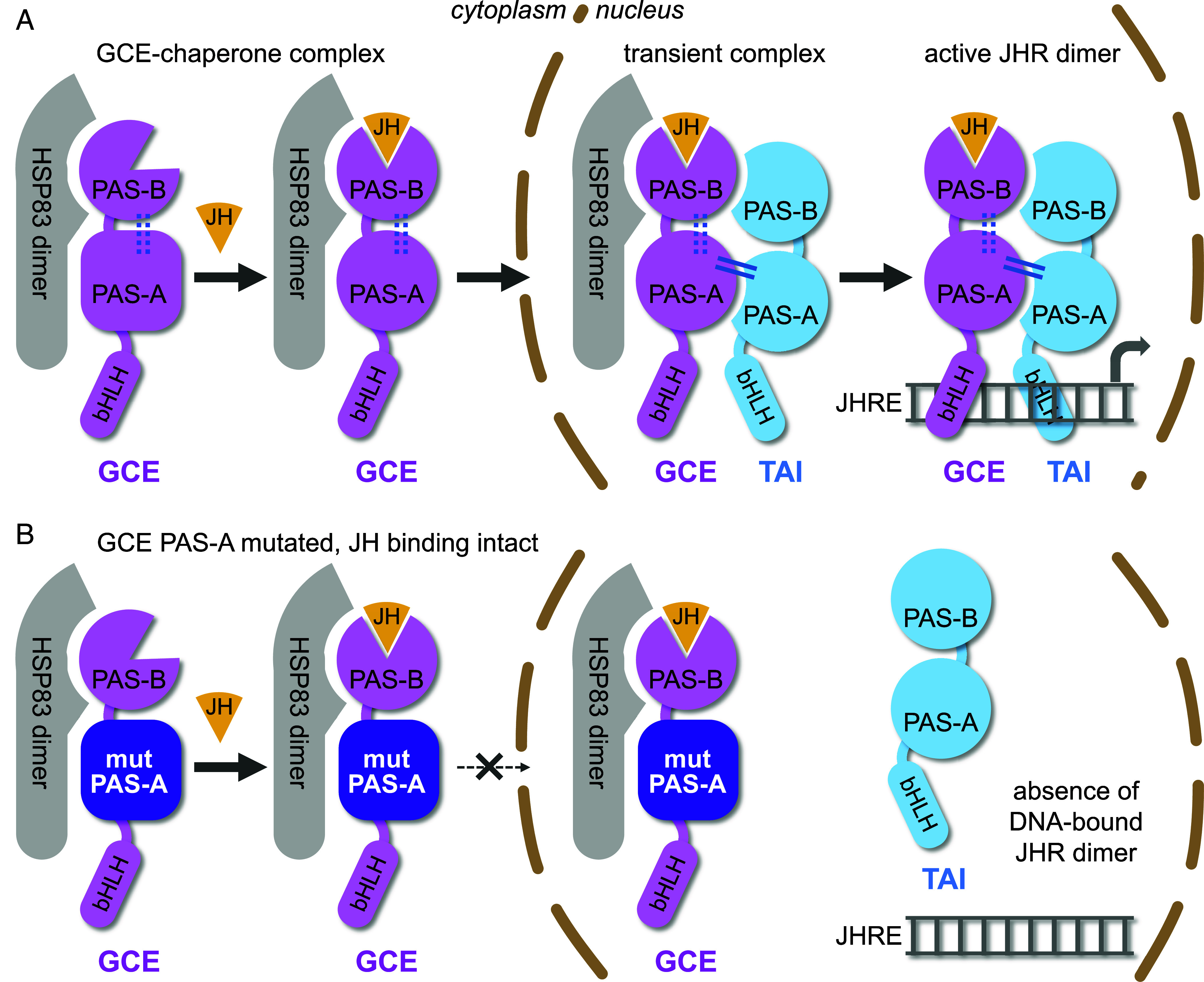
(*A*) A three-step model for JHR activation. Triggered by JH binding, conformational shifts in GCE (schematically depicted by PAS-B rotation and PAS-A shape change) lead to nuclear import of the GCE–chaperone complex, assembly of a hypothetical transient complex with TAI, and release of the JHR dimer from HSP83. Critical interdomain allosteric interactions are indicated by double lines. Whether the intramolecular interactions between the GCE PAS A and B domains (the dotted double lines) occur prior to JH binding or form during the JH-dependent complex transformation is unknown. (*B*) Without affecting JH binding, mutations of conserved charged residues at GCE PAS-A interfaces prevent the conformational changes, resulting in compromised nuclear import, augmented association with HSP83, and absence of GCE:TAI dimerization and DNA binding.

The intriguing finding was that the JH-binding capacity of the GCE PAS-B domain could be uncoupled from the protein–protein interactions of GCE and its transcriptional activity (Fig. 8). Thus, although the GCE variants R444A/R445A and E395A/D396A bound JH, they failed to respond to it by dimerizing with TAI and activating the *JHRE-luc* reporter. DNA-binding assays further corroborated that the loss of transactivation capacity resulted from the failure to form a GCE:TAI dimer capable of binding to the JHRE. Neither of the GCE double mutants was active when tested in vivo. Interestingly, the R444A/R445A mutation did not prevent formation of a GCE–GCE complex (*SI Appendix*, Fig. S8), indicating different modes of GCE interaction with TAI and with itself. The nature and significance of this GCE complex remains to be defined. Alanine substitution of R450, predicted to interact with E395 and D396 (Fig. 6), was another GCE PAS-A mutation causing severe reduction in the dimerization and transactivation capacities. Thus, apparently, removal of the conserved charged residues structurally altered GCE so that it could no longer dimerize with TAI.

The exact cause preventing the assembly of the mutated GCE variants into the active JHR complex is unclear. Besides impaired dimerization with TAI, the R444A/R445A, E395A/D396A, and R450A mutant proteins all displayed reduced dissociation from HSP83 in the presence of JH when compared to GCE^WT^. In theory, the PAS-A mutations could block GCE:TAI dimerization in two mutually nonexclusive ways: by trapping the liganded GCE in the HSP83 complex and/or by preventing it from binding to TAI directly. While our present data cannot resolve whether GCE must dissociate from HSP83 before it can engage TAI, a tentative answer may be inferred from a recent structural study on the AHR:ARNT complex (14). Their model posits that ligand binding to AHR, bound by HSP90 and the cochaperones XAP2 and P23 in the cytoplasm (13), induces nuclear import of the entire assembly, which is then joined by ARNT while AHR is still in contact with HSP90 and XAP2. ARNT is thought to eventually displace XAP2. After XAP2 and P23 leave the transient complex, the AHR:ARNT heterodimer is released from HSP90 and binds to DNA.

By analogy to AHR, we propose a three-step model for JHR signaling, in which i) a JH-triggered conformational change of GCE leads to nuclear import, ii) assembly of a transient complex including HSP83, GCE, and TAI, and iii) DNA binding of the GCE:TAI dimer (Fig. 8). Hence, prior release of GCE from HSP83 may not be prerequisite to GCE:TAI interaction. If that is the case, an intrinsic failure of the GCE PAS-A mutants to dimerize with TAI, rather than their nondissociation from HSP83, would appear to be the primary cause of disrupted JHR signaling. However, one has to keep in mind that MET/GCE and TAI are not orthologs of AHR and ARNT, respectively (8, 27), and that cochaperones similar to XAP2 and P23 have not been implicated in JHR activation. Therefore, the mechanisms of AHR and JHR activation may differ.

While structures of JHR complexes have yet to be resolved, models based on homology with known bHLH-PAS structures provided insights, primarily into the ligand-binding cavity of MET and GCE PAS-B domains (17, 18). In the present study, we have expanded the modeling to include the structured core of the PAS-A/B tandems of GCE and its partner TAI by using AlphaFold 3 and MD simulations. Based on these GCE:TAI models, the charged GCE PAS-A residues that have proven critical for JHR function locate to the putative domain interfaces. Initially, we focused on the Gβ strand, where mutating either R450 or the arginine pair 444/445 had strong detrimental effects on the JHR activity (Fig. 1).

Crystallography studies on bHLH-PAS complexes have revealed multiple inter- and intramolecular interfaces with individual interacting residues. The structures of HIF-2α:ARNT (30) and AHR:ARNT (31) dimers have implicated ARNT R260 (a position matching R444 in GCE; *SI Appendix*, Fig. S1) in interactions between the PAS-A domains of the respective partners. ARNT R266 (corresponding to R450 in GCE) interacts with HIF-2α PAS-B and is critical for stability of the HIF-2α:ARNT heterodimer (30). However, in the context of AHR:ARNT structures (14), ARNT R266 interacts with D217 within the same PAS-A domain (*SI Appendix*, Fig. S17), suggesting that this conserved residue may play alternative roles in different heterodimers across both subunit classes. Arginine residues homologous to GCE R450 engage in intramolecular links between the PAS-A and B domains within HIF-2α or the neuronal PAS protein 1 (NPAS1). Intriguingly, alanine substitution of these conserved arginines disrupts functional association of either HIF-2α or NPAS1 with ARNT (11, 30), illustrating the importance of intramolecular integrity for heterodimerization and activity of bHLH-PAS proteins. A similar scenario could be envisioned for interactions of GCE with TAI.

Our models have further predicted tight association of the GCE R450 with the highly conserved acidic residues (E395 and D396) within the presumptive Fα helix of the GCE PAS-A domain (Fig. 6), prompting us to test the GCE^E395A/D396A^ variant in functional assays. As already mentioned above, this double mutation has indeed proven deleterious for GCE performance in TAI dimerization, DNA binding, and *JHRE-luc* transactivation to an extent similar to the effect of the R450A substitution and as detrimental as that of R444A/R445A (cf. Figs. 1 and 7). These results demonstrate the utility of molecular modeling for successful prediction of unforeseen features that are critical for the receptor function.

MD simulations of the GCE:TAI dimer have suggested alterations in the complex structure resulting from the deleterious GCE PAS-A mutations (*SI Appendix*, Figs. S11, S15, and S16). The models predict structural shifts that disrupt the network of intra- and interdomain electrostatic interactions and lead to the partial solvent exposure of the PAS-A hydrophobic core. For instance, the E395A/D396A substitution causes the PAS-A Fα helix depart from the original R450 charge cluster (*SI Appendix*, Fig. S15). Conversely, the Fα helix swivels away from the Gβ strand when R450 is mutated (*SI Appendix*, Fig. S16). By replacing the salt bridges between the conserved charged residues with non-native interactions, these mutations likely disrupt the relative GCE domain disposition and thus affect stability of the JHR complex and/or the association of GCE with HSP83. This concept agrees well with the findings that the stable assembly of bHLH-PAS heterodimers requires structural integrity of the participating monomers (11, 14, 30, 33). Plausible roles of conserved residues equivalent to GCE R445 and R450 in solved structures of other PAS domain complexes in comparison with our GCE:TAI models are discussed in detail in *SI Appendix*, Figs. S17–S19 and *Supplementary Discussion*.

In summary, this study has uncovered vital role of evolutionarily conserved charged residues at the interfaces of the GCE PAS-A domain in regulating JH-dependent JHR assembly and signaling without blocking its ligand binding capability. Thus, while ligand binding to PAS-B domain is required to trigger JHR signaling, it is not sufficient and another step involving interdomain interactions is necessary for nuclear transport and the subsequent dimerization. This is summarized in a plausible mechanistic model (Fig. 8) where we propose that PAS-A and B domains are allosterically coupled via the conserved domain interface through which conformational changes upon JH binding are transduced.

While the JHR proteins MET/GCE are unique to protostomes, we conclude that structural integrity and function of the JHR and of diverse mammalian bHLH-PAS complexes alike depend on similar intra- and interdomain links formed by residues located in their PAS-A domains. Resolving JHR structures is needed to understand exactly how conformational changes induced by JH activate JHR signaling.

## Materials and Methods

### Compounds, Constructs, and Cell Cultures.

Racemic 10*R,S*-JH III was from Sigma-Aldrich and the stocks were diluted in ethanol. The radiolabeled 10*R,S*-[^3^H]-JH III was de novo synthesized and tritiated (37). *S*-methoprene (VUOS Pardubice, Czechia) was dissolved in dimethyl sulfoxide (DMSO).

The DNA sequence encoding the full-length *D. melanogaster* GCE protein (NCBI entry NP_001188593.1) was subjected to site-specific PCR mutagenesis using the primers described in *SI Appendix*, Table S1 and was verified by sequencing. The full-length GCE variants were cloned to *pIEx-4* (Novagen) for the *JHRE-luc* dual luciferase reporter assay, *pACT* (Promega) for the two-hybrid assay, *pK-Myc-C2* for expression in the HEK293 cells, and to *pUAST-attB* for generating transgenic *Drosophila*. N-terminally truncated (G278-D959) GCE variants (Fig. 1*A*) were cloned to the *pK-Myc-C2* and *pBiT1.1-N[TK/LgBiT]* (Promega) vectors for the JH-binding and NanoBiT assays, respectively. For the EMSA, either GCE (G278-D959) or full-length versions were used. *D. melanogaster* TAI (NP_001245949.1) was C-terminally truncated at S740 and expressed from the vectors *pBIND* (Promega) (36) for the two-hybrid assay, *pEGFP-C2* (Clontech) for co-IP, and *pK-Myc-C2* for the EMSA, respectively. The previously described vector *pBiT2.1-N[TK/SmBiT]* (Promega) expressing the HSP83 protein from *T. castaneum* (NP_001300806.1) was used for the NanoBiT assay (24).

*D. melanogaster* Kc167 embryonic cells were grown at 26 °C in Schneider’s *Drosophila* Medium (Gibco, Life Technologies) supplemented with 10% fetal bovine serum (Sigma-Aldrich), 100 U/mL penicillin, and 100 μg/mL streptomycin (Life Technologies). The human embryonic kidney (HEK293) and CHO cell lines were cultured in Dulbecco’s Modified Eagle’s Medium (DMEM; Life Technologies) containing 10% fetal bovine serum (Sigma-Aldrich), 100 U/mL penicillin, and 100 μg/mL streptomycin at 37 °C in humidified atmosphere with 5% CO_2_. All cell transfections were performed using FuGENE HD (Promega).

### Dual Luciferase Reporter Assay.

The *JHRE-luc* firefly luciferase reporter was used as previously described (7) to assess the effect of JH III in the *Drosophila* Kc167 cell line transiently expressing WT and mutated variants of GCE. Cells in 24-well plates were transfected with *JHRE-luc* reporter (125 ng/well), *Renilla* luciferase and a GCE construct (each 50 ng/well). The endogenous GCE was suppressed by adding 1 µg/well of double-stranded RNA targeting the native *gce* mRNA, or *egfp* dsRNA for control. After 36 h, cells were incubated with 100 nM JH III or the vehicle (ethanol) for an additional 10 to 12 h. Luminescence was measured with the Dual-Luciferase assay (Promega) using the Orion II microplate luminometer (Berthold). Reporter luciferase activity was normalized to *Renilla* luminescence.

### Two-Hybrid and Split Luciferase (NanoBiT) Assays.

Ligand-dependent dimerization of the *Drosophila* JHR subunits, GCE and TAI (M1-S740), fused to the VP16 transactivation and the Gal4 DNA-binding domain, respectively, was assessed in transiently transfected HEK293 cells using the CheckMate Mammalian Two-Hybrid System (Promega) as described (16). The NanoBiT (NanoLuc luciferase complementation assay) (40) was performed in CHO cells as described (24). Briefly, cells were seeded semiconfluent on solid white, 96-well plates (#3917, Corning), and 24 h later transfected with plasmids encoding the *T. castaneum* HSP83 with the N-terminal small-bit (Sm) NanoLuc tag and either WT or mutated GCE variant N-terminally tagged with the NanoLuc large-bit (Lg), 50 ng of each plasmid per well. After another 24 h, cells were equilibrated for 15 min in serum-free DMEM containing 20 mM HEPES (pH 7.2) and supplemented with the Nano-Glo Live Cell Substrate (Promega), and the baseline was measured for 20 min. After adding 1 μM JH III or ethanol, luminescence was monitored for further 20 min using the Orion II (Berthold) microplate reader. The luminescence levels for each well were normalized to the baseline.

### JH Binding Assay.

Binding of [^3^H]-JH III (25.7 Ci/mmol) (37) was assessed using the dextran-coated charcoal method as previously described (4) with the WT and mutated GCE proteins translated in vitro using the TnT Quick T7 Coupled system (Promega). The GCE-bound [^3^H]-JH III was quantified by scintillation counting using the Hidex 300 SL liquid scintillation counter (HIDEX, Finland).

### *Drosophila* Genetics.

Flies carrying transgenic constructs for the WT or mutated GCE proteins were generated as described previously (7). All transgenes in the *pUASTattB* vector were inserted into the *attP2* landing site on the third chromosome using the bacteriophage *ϕC31* integrase (41). The constructs were injected to *D. melanogaster* embryos of the host genotype *y w P{nos-ϕC31\int.NLS}X; P{CaryP}attP2* (Fly Facility, University of Cambridge, United Kingdom). Expression of the transgenic proteins was induced using the Gal4/UAS system (42) with one of the following drivers: the ubiquitous *armadillo* (*arm-Gal4*) for genetic rescue, *patched* (*ptc-Gal4*) or *eyeless* (*ey-Gal4*) for expression in the larval salivary glands or the eye imaginal discs, respectively. All driver lines were from the Bloomington *Drosophila* Stock Center (Bloomington, IN). For genetic rescue experiments, the *UAS-gce* transgenic males were crossed with *Met^27^ gce^2.5k^/FM7c; arm-Gal4* females and viable mutant *Met^27^ gce^2.5k^/Y* adult male progeny were scored against their balanced *FM7c/Y* siblings as a measure of the GCE functionality; equal number of the mutant and balanced emerged males was considered 100% rescue of the nonconditionally lethal *Met^27^ gce^2.5k^/Y* males. The data from multiple crosses were evaluated by multiple comparisons using Brown–Forsythe and Welch ANOVA tests using Prism 9 (GraphPad).

### Immunostaining of Larval Tissues and HEK293 Cells.

Dissected salivary glands or imaginal discs from third instar wandering *Drosophila* larvae were stained as described (43) with a rat anti-GCE polyclonal serum, generously provided by Claude Desplan (New York University, NY) diluted 1:500, followed by staining with a Cy3-conjugated anti-rat antibody (1:500, Jackson ImmunoResearch). Localization of Myc-tagged WT and mutated GCE protein variants in the HEK293 cells was detected as described (23). The cells were stained with the 9E10 mouse antibody against the Myc epitope (1:500; Invitrogen), followed by Cy5-labeled anti-mouse antiserum (1:500; Jackson ImmunoResearch) 36 h after transfection with the appropriate *gce* expression constructs and 1-h incubation with 1 µM JH III or vehicle (ethanol) alone. Stained tissues and cells were mounted in the VectaShield medium with DAPI (Vector Laboratories, Newark, CA), and images were captured using the FluoView FV3000 confocal microscope (Olympus). The nucleo-cytoplasmic distribution of GCE in the HEK293 cells was assessed using ImageJ (NIH). The nuclear-to-total signal ratio was calculated from integrated raw intensity for individual cells (50 to 141 cells in total for each variant) from single confocal slices. The statistical significance of the observed differences was evaluated by one-way ANOVA, Dunnett’s multiple comparisons test using Prism 9 (GraphPad).

### Immunoprecipitation.

WT or mutated variants of 3xFlag-Myc-GCE in *pK-Myc-C2* and EGFP-TAI (M1-S740) in *pEGFP-C2* (Clontech) were transfected in the HEK293 cells grown on 12-well plates. After 40 h, the cells were treated for 2 h with 1 μM JH III or ethanol vehicle alone. The cells were lysed in TBS buffer (50 mM Tris-HCl, pH 7.4, 150 mM NaCl, 1 mM EDTA) containing 0.2% Tween-20, Complete EDTA-free protease inhibitors (Roche), and supplemented with 1 µM JH III as appropriate. The JHR proteins were pulled down by 1-h incubation with GFP-Trap (Chromotek) or anti-Flag M2 (Sigma-Aldrich) magnetic beads, to capture the complexes via EGFP-TAI or 3xFlag-Myc-GCE, respectively. After three washes with TBS, the beads were incubated in the LDS sample buffer (Thermo Scientific) for 10 min at 75 °C. Eluted proteins and aliquots from the respective inputs were analyzed by immunoblotting using rabbit anti-GFP (1:1,500, Invitrogen) and mouse anti-Myc 9E10 (1:2,000, Invitrogen) or mouse anti-Flag tag FG4R monoclonal (1:1,000, Invitrogen) antibodies. Signal was detected with appropriate HRP-conjugated secondary antibodies (Life Technologies) and the SuperSignal West Femto Maximum Sensitivity substrate (Thermo Scientific).

### Fluorescence-Based EMSA.

The ability of GCE and TAI proteins to dimerize and bind target DNA in vitro was tested using an upstream *k-JHRE* sequence originating from the immediate JH-response gene *Kr-h1* of *T. castaneum* (39). The fluorescently labeled double-stranded *k-JHRE* probe was prepared by annealing 5′-Cy-GTACTTCGCCTC**CACGTG**CCGTTTCAACAC with the Cy-5-labeled complementary oligonucleotide; the essential E-box is in bold. Competition with unlabeled WT *k-JHRE* oligonucleotide (5′-TCGCCTC**CACGTG**CCGTTTCA) or a mutated DNA lacking the E-box (5′-TTCGCCTCACATGTCCGTTTCA) served to assess the specificity of the interaction. The GCE (either full-length or N-terminally truncated at G278) and TAI (M1-S740) proteins were expressed either individually or in combination from the *pK-Myc-C2* vector (each plasmid 500 ng DNA) using the TnT Quick T7 Coupled in vitro transcription/translation system (Promega). The reaction lysates (2 µL aliquots) were incubated for 30 min at room temperature with the fluorescent probe (5 pmol) in the presence of 8.5 µM methoprene or the vehicle (ethanol), in a binding buffer [20 mM Tris-HCl (pH 7.8), 50 mM NaCl, 5 mM MgCl_2_, 5 mM DTT, 10% glycerol, 500 ng/µL bovine serum albumin (BSA) and 50 ng/µL poly(dAdT)]. Competition assays also included 10-fold excess of the unlabeled double-stranded oligonucleotide. The protein–DNA complexes were resolved by electrophoresis on native 6% polyacrylamide gels in Tris-borate-EDTA buffer (pH 8.3), and the Cy5 signal was detected directly in the wet gels using the ChemiDoc imaging system (Bio-Rad).

### Molecular Modeling.

The initial GCE:TAI heterodimer model was generated with AlphaFold 3 for the minimal functional fragments of GCE and TAI. In order to examine the effects of mutations, these models were further trimmed to encompass only the structured PAS domains and their loops and connecting helices. This model was subjected to 3 µs MD relaxation in Gromacs 2023.1 (44) using AMBER99SB-ILDN force field (45) as described previously (18). The final frame was used as the starting structure for introducing mutations and for control simulations of the WT dimer. Distributions of distance were computed in Gromacs.

## Supplementary Material

Appendix 01 (PDF)

## Data Availability

Study data are included in the article and/or *SI Appendix*.
