# Supplementary material for: Juvenile hormone receptor relies on conserved domain interfaces for dimerization-dependent activity
Source: Proc Natl Acad Sci U S A. 2026 Aug 6;123(32):e2605755123. doi: 10.1073/pnas.2605755123 (PMC13462165; doi:10.1073/pnas.2605755123)
Supplement: Supplementary file 1 [file pnas.2605755123.sapp.pdf]

Supporting Information

for

Juvenile hormone receptor relies on conserved domain interfaces for dimerization-dependent activity

Lenka Bittova[1], Sarka Tumova[1], Mykola Yatsenko[1,2], Raveendra Babu Mokhamatam[1,3], Roman Tuma[2,*] and Marek Jindra[1,2,*]

[1] Institute of Entomology, Biology Center of the Czech Academy of Sciences, Ceske Budejovice 37005, Czech Republic
[2] Faculty of Science, University of South Bohemia, Ceske Budejovice 37005, Czech Republic
[3] Preagon Biotech, s.r.o., Zahornice 28903, Czech Republic

Corresponding authors:

Marek Jindra, e-mail: jindra@entu.cas.cz

Roman Tuma, e-mail: rtuma@prf.jcu.cz

This PDF file includes:

Supplementary Figures S1 to S19
Supplementary Tables S1 and S2
Supplementary Discussion
References

Supplementary Figures

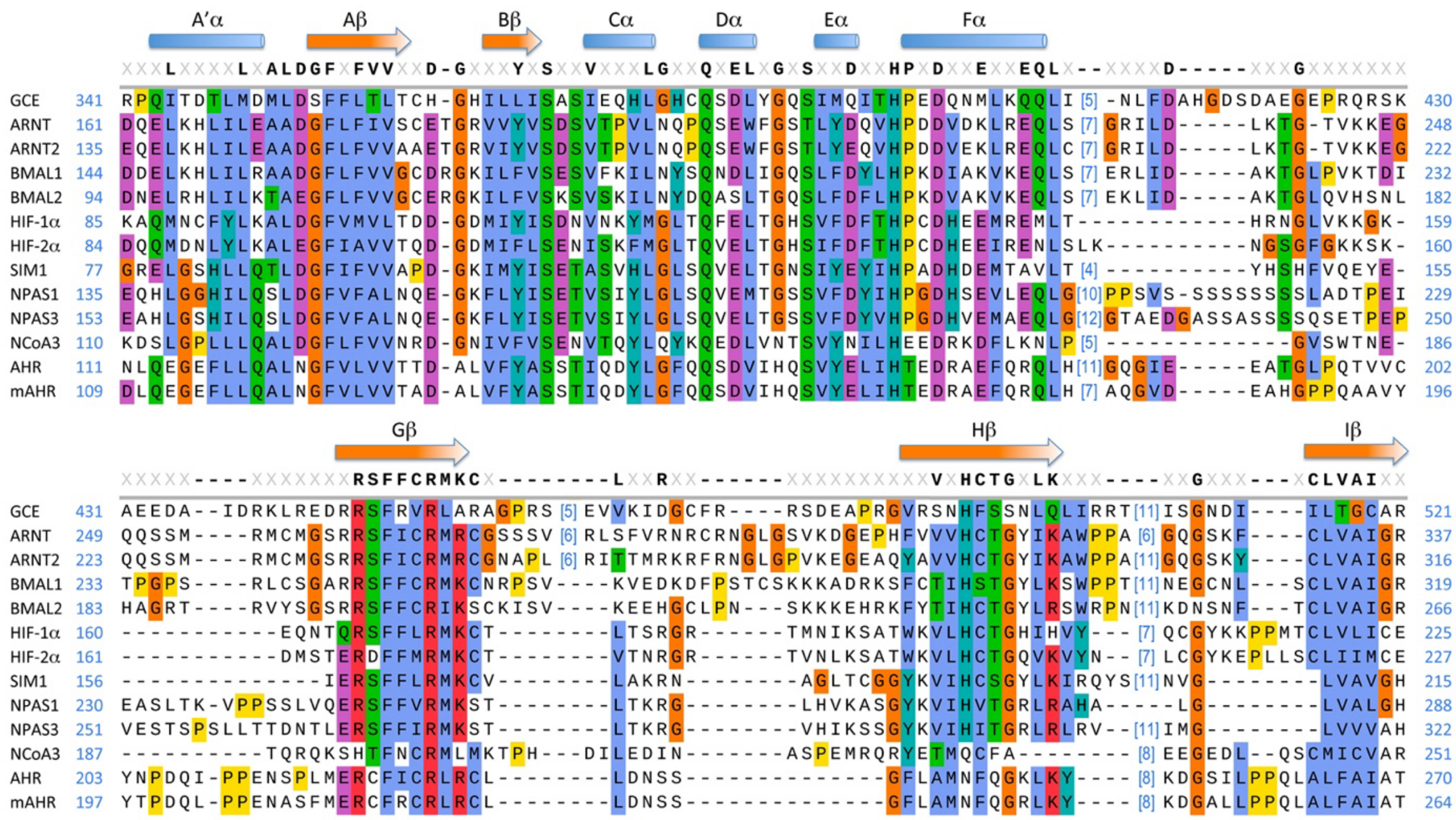

Fig. S1. Homology between the PAS-A domain of *Drosophila* GCE and seven types of selected mammalian bHLH-PAS proteins. The α-helices and β-strands are denoted above the consensus sequence based on crystal structures of ARNT and HIF-2α (1). The multiple sequence alignment was generated using the MAFFT tool of the EMBL (2). Numbers indicate amino acid positions; numbers in brackets are the numbers of residues not displayed. Except for *Drosophila* GCE and murine AHR (mAHR), the proteins are human. The NCBI accession numbers for the sequences are as follows: *Drosophila* GCE, NP_001188593.1; ARNT, XP_047276675.1; ARNT2, NP_055677.3; BMAL1, BAA19935.1; BMAL2, BAB01485.1; HIF-1α, KAI2571528.1; HIF-2α, NP_001421.2; SIM1, NP_001361698.1; NPAS1, NP_002508.2; NPAS3, KAI2570913.1; NCoA3, NP_001167559.1; AHR, BAA03857.1; mAHR, NP_038492.1.

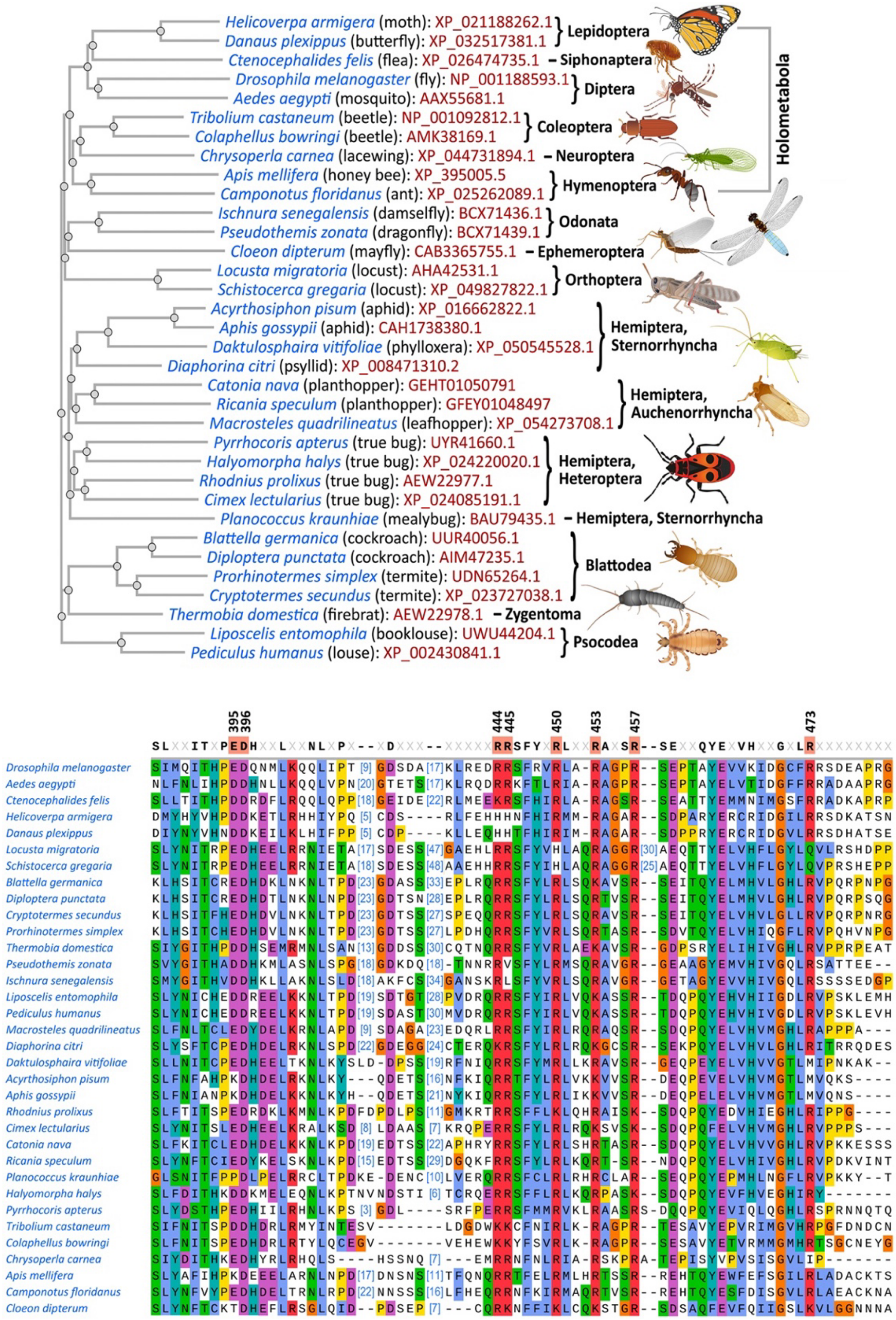

Fig. S2. Insect species representing six holometabolous and seven hemimetabolous orders (top) chosen for alignment of GCE/MET protein orthologs (the NCBI accession numbers are indicated). Conservation within the C-terminal portion of the PAS-A domain encompassing the predicted Fα helix and Gβ strand (see Fig. S1). The multiple sequence alignment was generated using the MAFFT tool of the EMBL (2). Numbers in brackets are the numbers of residues not displayed. The conserved glutamic acid (E395), aspartic acid (D396), and arginine (R) residues (top, highlighted in orange) in *Drosophila* GCE were mutated to alanine in this study.

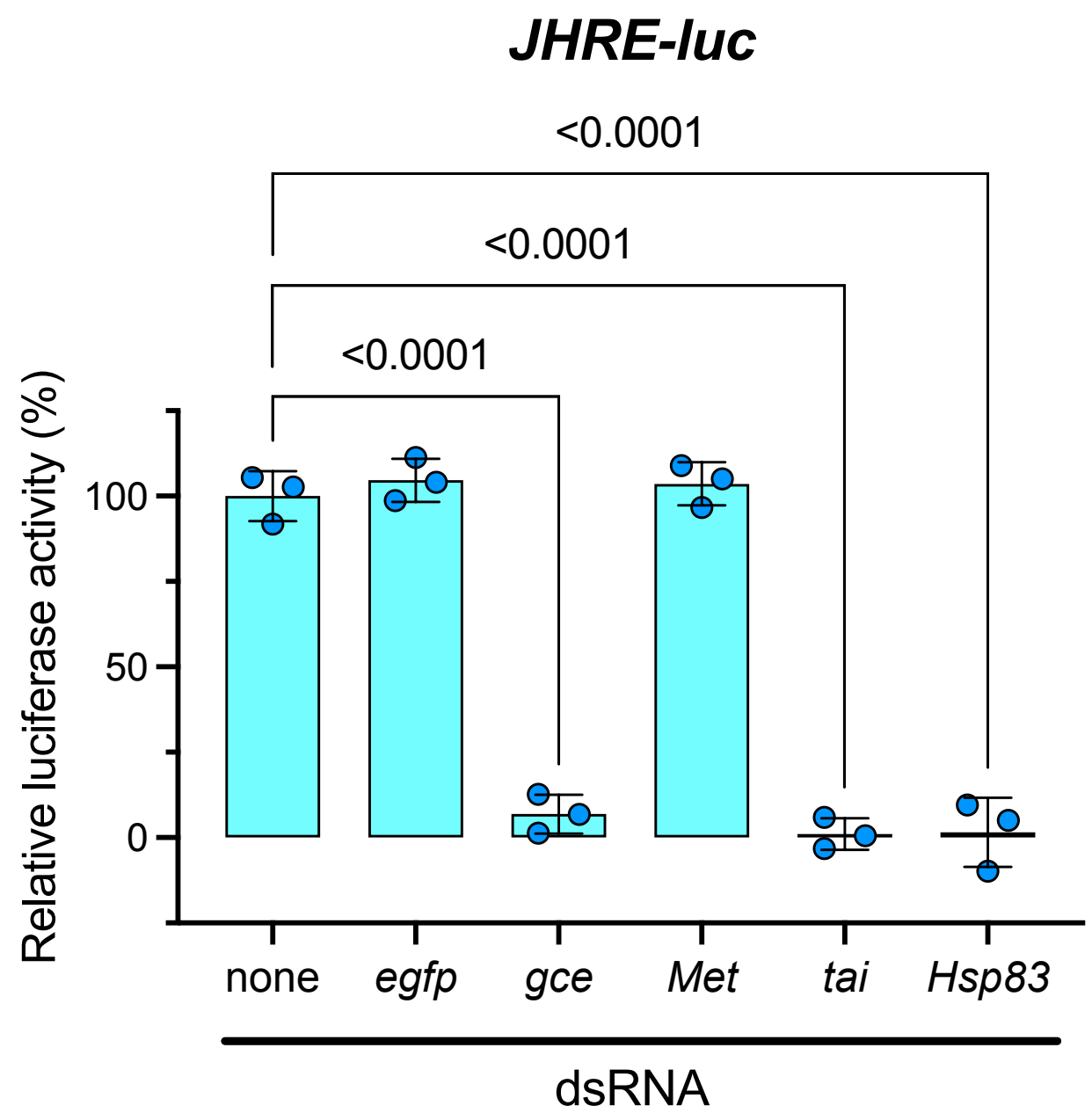

Fig. S3. Effect of RNAi knockdown on JH-dependent activation of the *JHRE-luc* transcriptional reporter. A *Drosophila* Kc167 cell line stably transfected with the *JHRE*-driven firefly luciferase reporter (3) was subjected to RNAi-mediated knockdown of the indicated endogenous genes using double-stranded RNA (dsRNA); control treatments were with water alone (none) and *egfp* dsRNA. The dsRNA was synthesized *in vitro* from *D. melanogaster* cDNA using T7 RNA polymerase and primers listed in Table S2. The cells (8 x 10^4 cells in 20 µl of serum-free Schneider's *Drosophila* medium) were mixed with 0.2 µg of dsRNA in 10 µl of water per well in a 96-well plate and incubated at room temperature for 30 min. Schneider's *Drosophila* medium supplemented with 10% fetal bovine serum was added (70 µl per well) and the cells were kept at 27°C for 3 days. After treatment with 10 µM JH III, the cells were incubated for another 8 hours and then lysed in 50 µl per well of the Bright-Glo Luciferase Assay System (Promega) at room temperature for 10 min before measuring luminescence. Cell viability was determined for all dsRNA treatments in parallel using the CellTiter-Glo Luminescent Assay (Promega). Cell viability remained between 96-100% across all treatments. Data are plotted as mean ± SD relative to the luciferase activity measured with solvent alone (0%) and with 10 µM JH III in the absence of dsRNA (100%) and normalized to cell viability. *P* values (one-way ANOVA) are indicated for significant differences from the control without dsRNA.

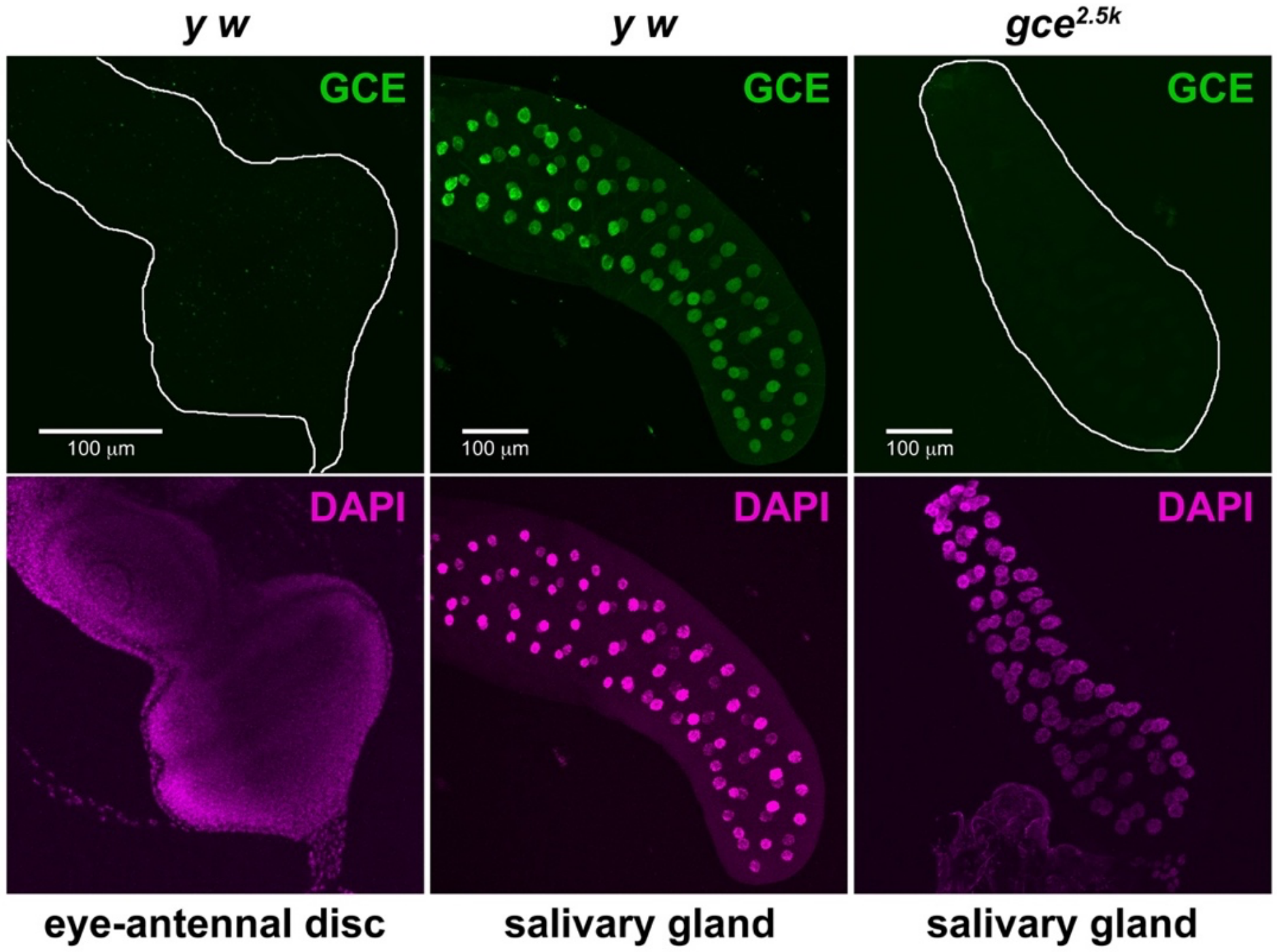

Fig. S4. The eye-antennal imaginal disc and salivary glands from third-instar *Drosophila* larvae stained with the anti-GCE antibody and counterstained with DAPI. The specific GCE signal seen in the control (*y w*) salivary gland (middle, green) was undetectable either in the *gce*$^{2.5k}$ null mutant gland (right) or in the control (*y w*) imaginal disc (left); the shape of the organs is outlined. The images are Z-stacks of multiple confocal slices. The scale bars are 100 µm in all panels.

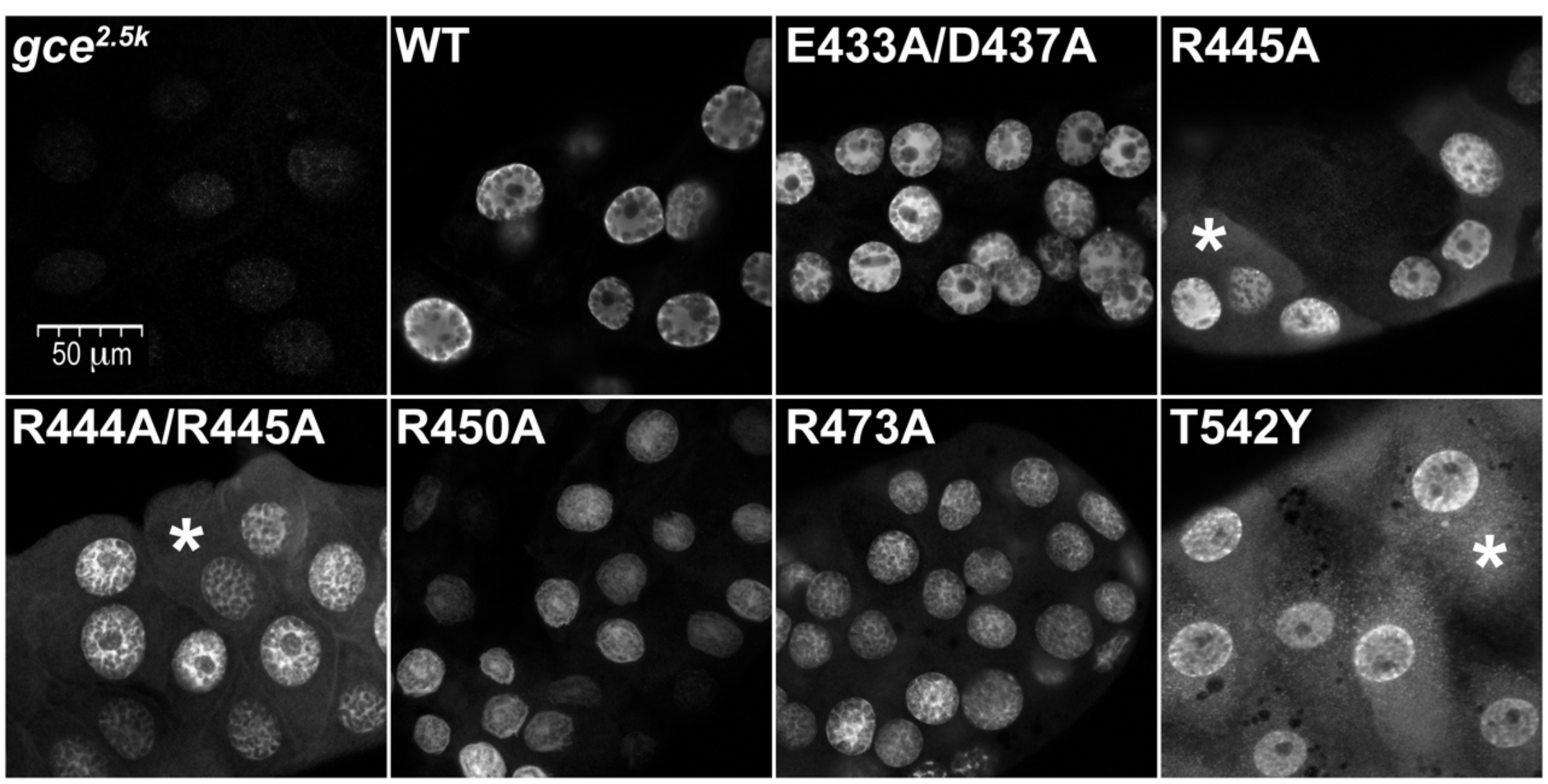

Fig. S5. Localization of selected GCE variants in the larval salivary glands. The wild-type (WT) and mutated transgenic GCE proteins were detected using the anti-GCE antibody in the *gce*$^{2.5k}$ mutant background lacking the endogenous GCE protein. Besides the predominant nuclear staining, the variants with partially (R445A) or completely (R444A/R445A and T542A) inactivating mutations showed an appreciable cytoplasmic presence (asterisks). The localization varied among individual larvae and cells; representative samples are shown. The images are confocal Z-stacks; the scale bar (50 µm) applies to all samples.

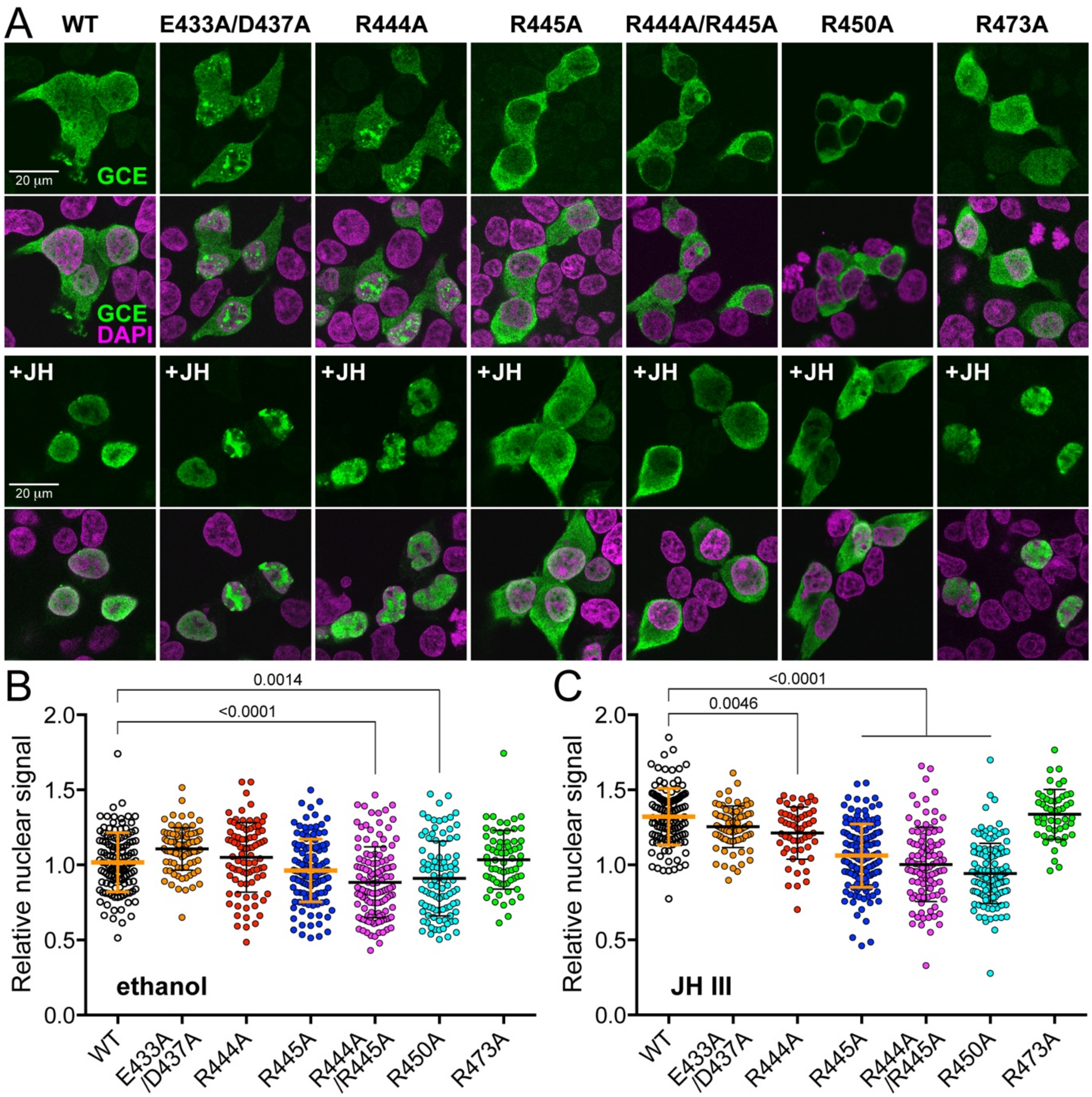

Fig. S6. Effects of mutations and JH on the cellular localization of GCE proteins. (*A*) The WT and indicated mutant variants of GCE, all carrying an N-terminal Myc-tag, were transiently expressed in HEK293 cells. After 1-hour incubation with 1 µM JH III (+ JH) or ethanol, cells were stained with anti-Myc antibody (green) and DAPI (magenta). The images are single confocal slices; the scale bar (20 µm) applies to all samples. (*B,C*) Cellular localization of GCE was assessed as integrated pixel densities in confocal slices from stained transfected cells; each dot represents the nuclear-to-total signal ratio for an individual cell, either from the vehicle control (*B*) or treated by JH III (*C*). The R444A/R445A and R450A mutations most prominently reduced nuclear localization in both cases. JH III strongly induced nuclear localization of the WT, E433A/D437A and R473A variants, although all GCE variants except for R450A displayed at least a minor shift towards the nucleus (see main text, Fig. 3). The statistical significance was determined by one-way ANOVA Dunnett's multiple comparisons test. All data in (*B,C*) are presented as mean ± SD; *P* values are indicated only for significant differences from the control (WT).

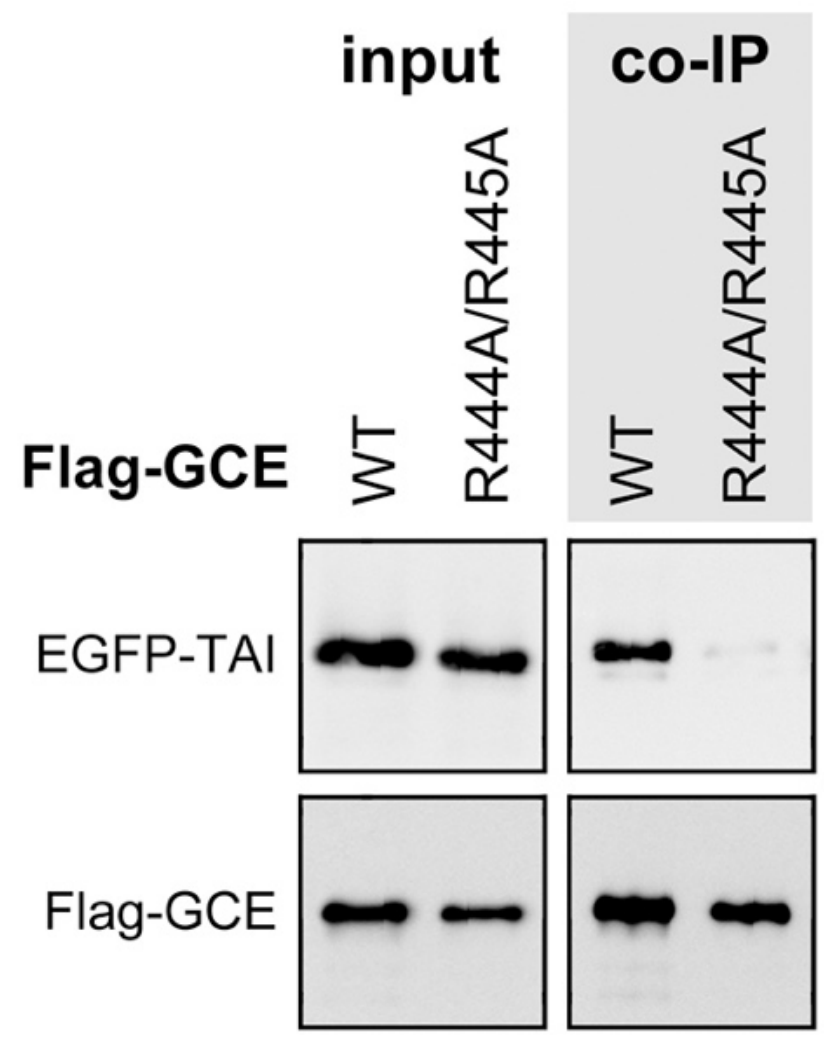

Fig. S7. Co-immunoprecipitation of GCE and TAI proteins. The 3xFlag-GCE WT or R444A/R445A variants were co-expressed with EGFP-TAI in HEK293 cells, treated with 1 μM JH III for 1 h prior to cell lysis. The complexes were pulled down using anti-Flag M2 beads and the interacting proteins were detected by immunoblotting with anti-EGFP (co-IP) and anti-Flag antibodies. The input panel represents 1.5% of the starting lysate; 15% of the immunoprecipitated material was loaded. Relative to GCE^{WT}, the interaction of $GCE^{R444A/R445A}$ with TAI was greatly reduced.

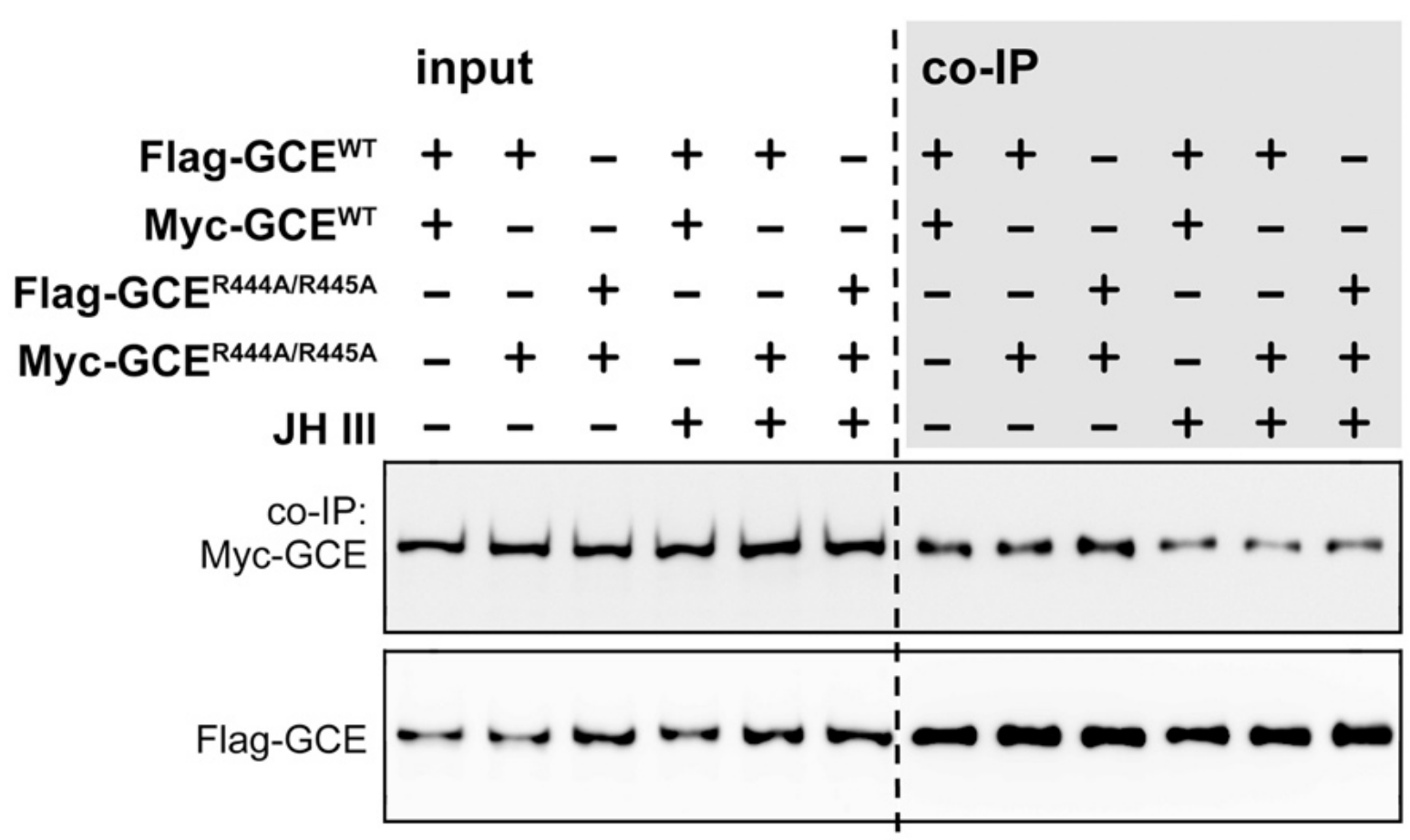

Fig. S8. Co-immunoprecipitation of a GCE:GCE complex. The 3xFlag- and Myc-tagged GCE WT or R444A/R445A variants were co-expressed in HEK293 cells, treated with 1 μM JH III or vehicle alone for 1 h prior to cell lysis. The complexes were pulled down using anti-Flag M2 beads and the GCE proteins were detected by immunoblotting with anti-Myc (co-IP) and anti-Flag antibodies. The input panel represents 1.4% of the starting lysate; 16% of the immunoprecipitated material was loaded. The R444A/R445A mutation had no effect on the GCE:GCE interaction, which was partially reduced in the presence of JH III.

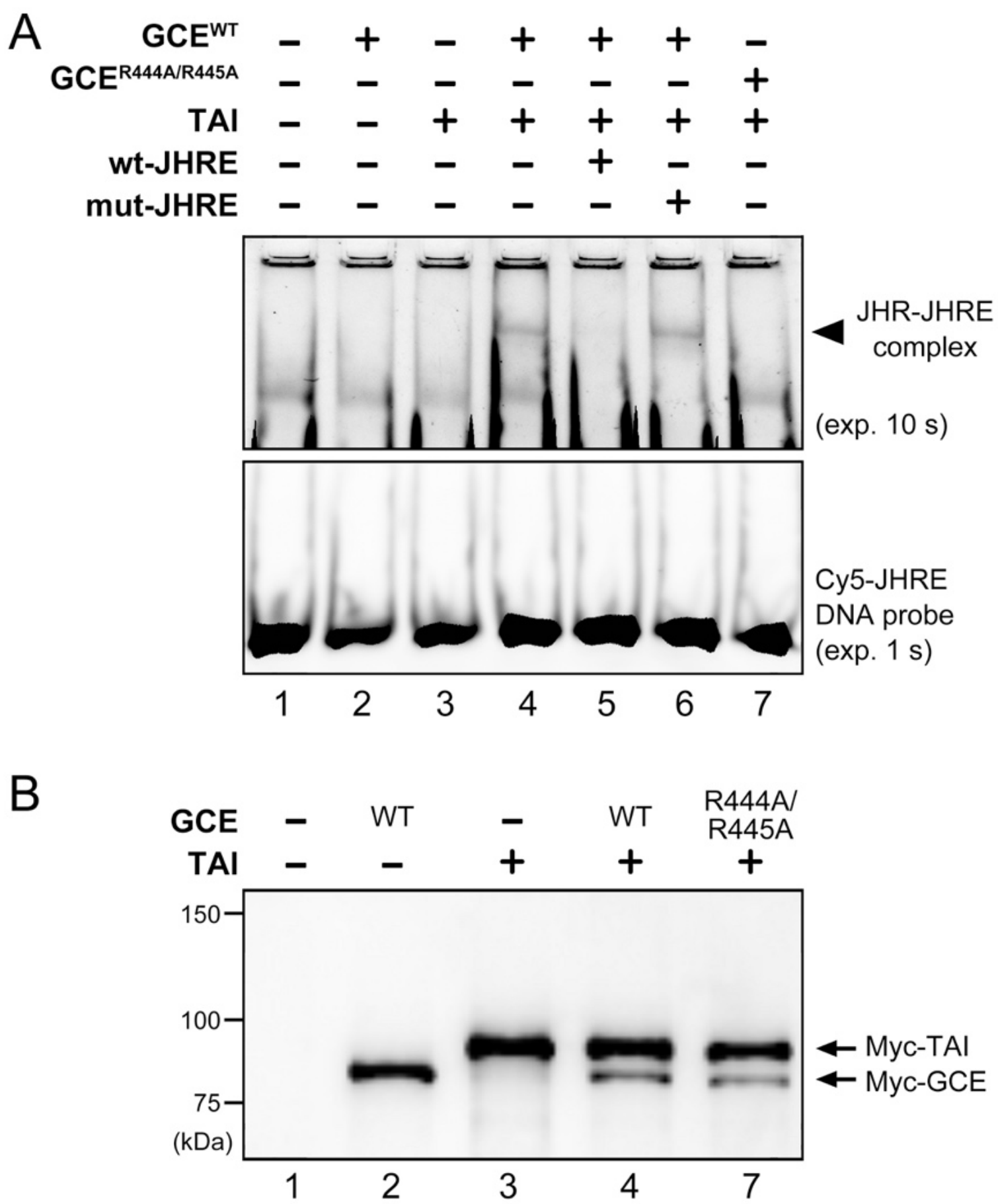

Fig. S9. (*A*) Electrophoretic mobility shift assay (EMSA). The *in-vitro* translated GCE and TAI proteins were incubated with the Cy5-labeled wt-JHRE oligonucleotide in the presence of 8.5 µM methoprene. Both GCE and TAI are required to form a complex with the JHRE DNA (arrowhead), as neither protein alone produced the retarded band (*cf*. lanes 2 and 3 with lane 4). The amount of the complex was reduced by adding 10-fold excess of unlabeled wt-JHRE (lane 5) but not the mutated JHRE oligonucleotide (lane 6). The GCE$^{R444A/R445A}$ mutant was unable to form the JHRE-bound complex (lane 7). The GCE and TAI proteins were expressed either separately (lanes 2 and 3) or together (lanes 4-7); lane 1 contained mock-translation lysate. The bottom part of the same gel shows the unreacted Cy5-JHRE DNA probe, detected with shorter exposure. (*B*) Immunoblotting of the *in-vitro* translation lysates confirms input of the GCE and TAI proteins in the EMSA reactions. The lane numbers match those of the EMSA reactions in (*A*).

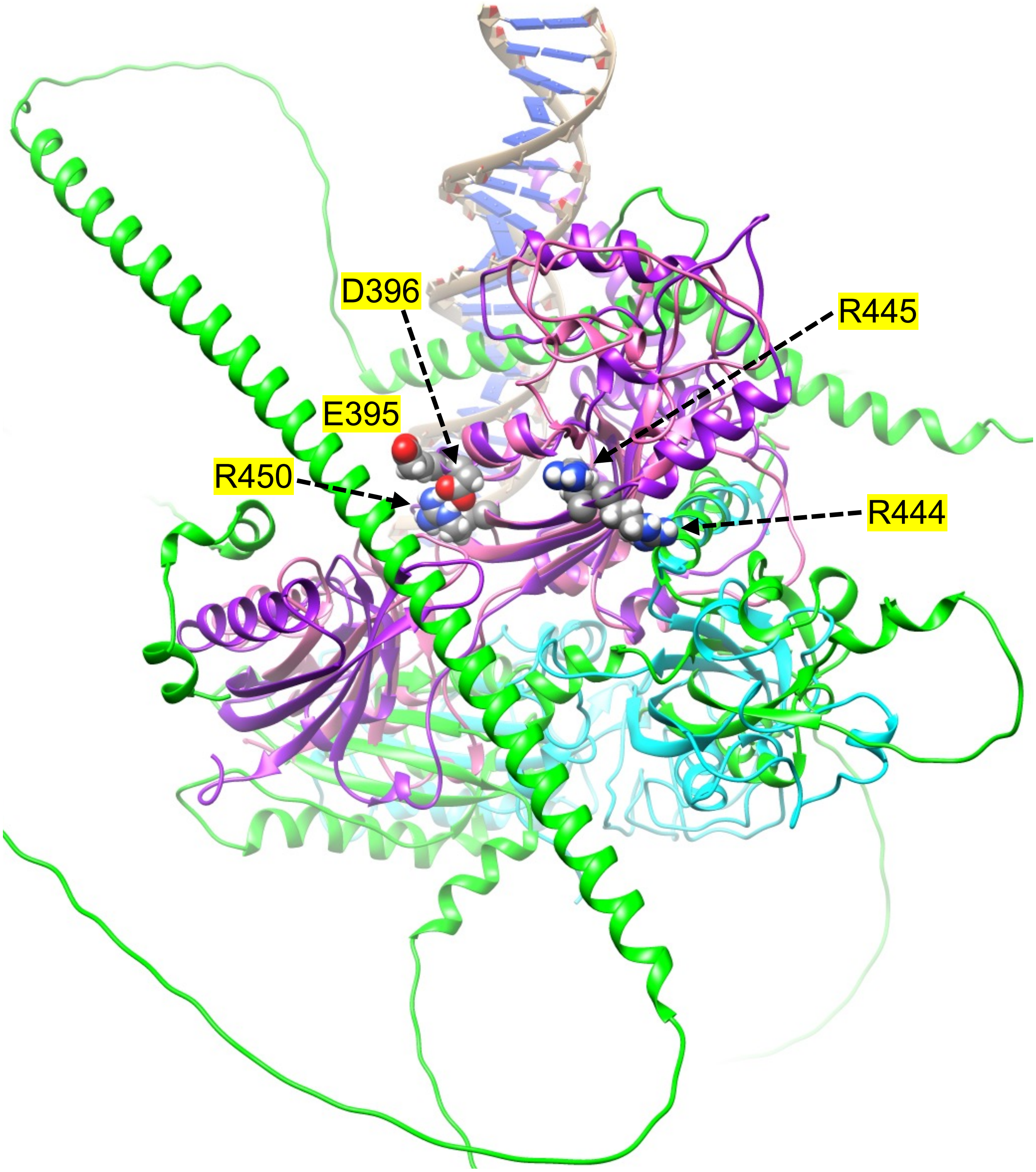

Fig. S10. Initial AlphaFold 3 model of the DNA-bound GCE (magenta) : TAI (green) complex overlaid with the structured PAS-A/B core (GCE, pink; TAI, cyan) after 3-μs all-atom MD relaxation. The disordered C termini were removed for clarity. Selected GCE PAS-A charged residues whose replacement with alanine causes strong signaling defects are shown in spacefill representation.

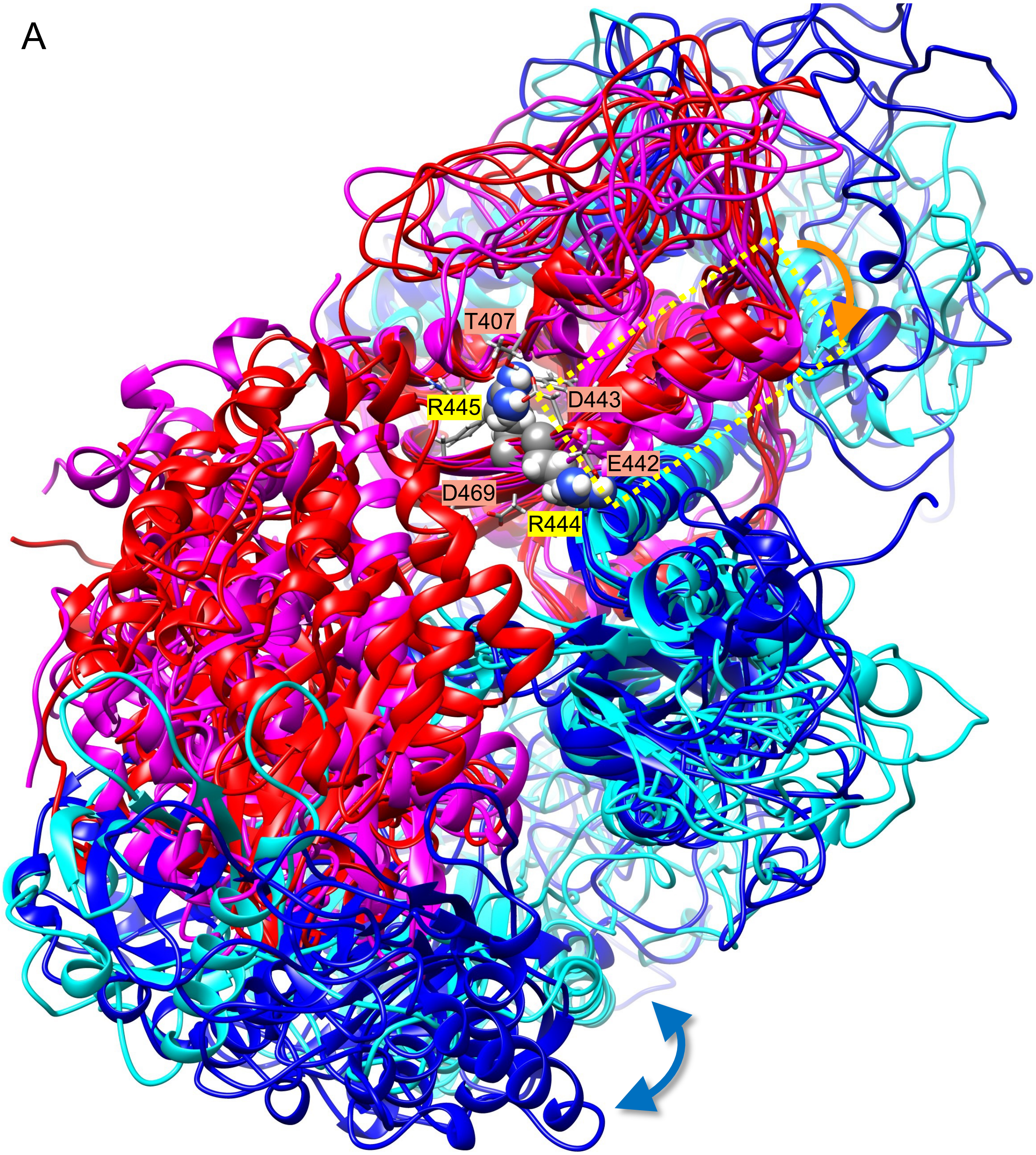

Fig. S11. Structural consequences of the GCE R444A/R445A double mutation. The wild-type GCE (red) : TAI (blue) PAS-A heterodimer is compared with mutated GCE (magenta) : TAI (cyan) after 1-μs all-atom MD simulation from the same starting configuration. (*A*) Comparison of prevailing conformations within the stationary phase of simulation (stable RMSD) revealed local structural departure in the position of an α-helix that is adjacent to the mutated site (dashed yellow box) as indicated by the orange arrow. This change was accompanied by global repositioning the TAI PAS-B domain (blue arrow). The mutated residues (yellow labels) are shown in spacefill while their interacting partners (orange labels) are shown in stick representation.

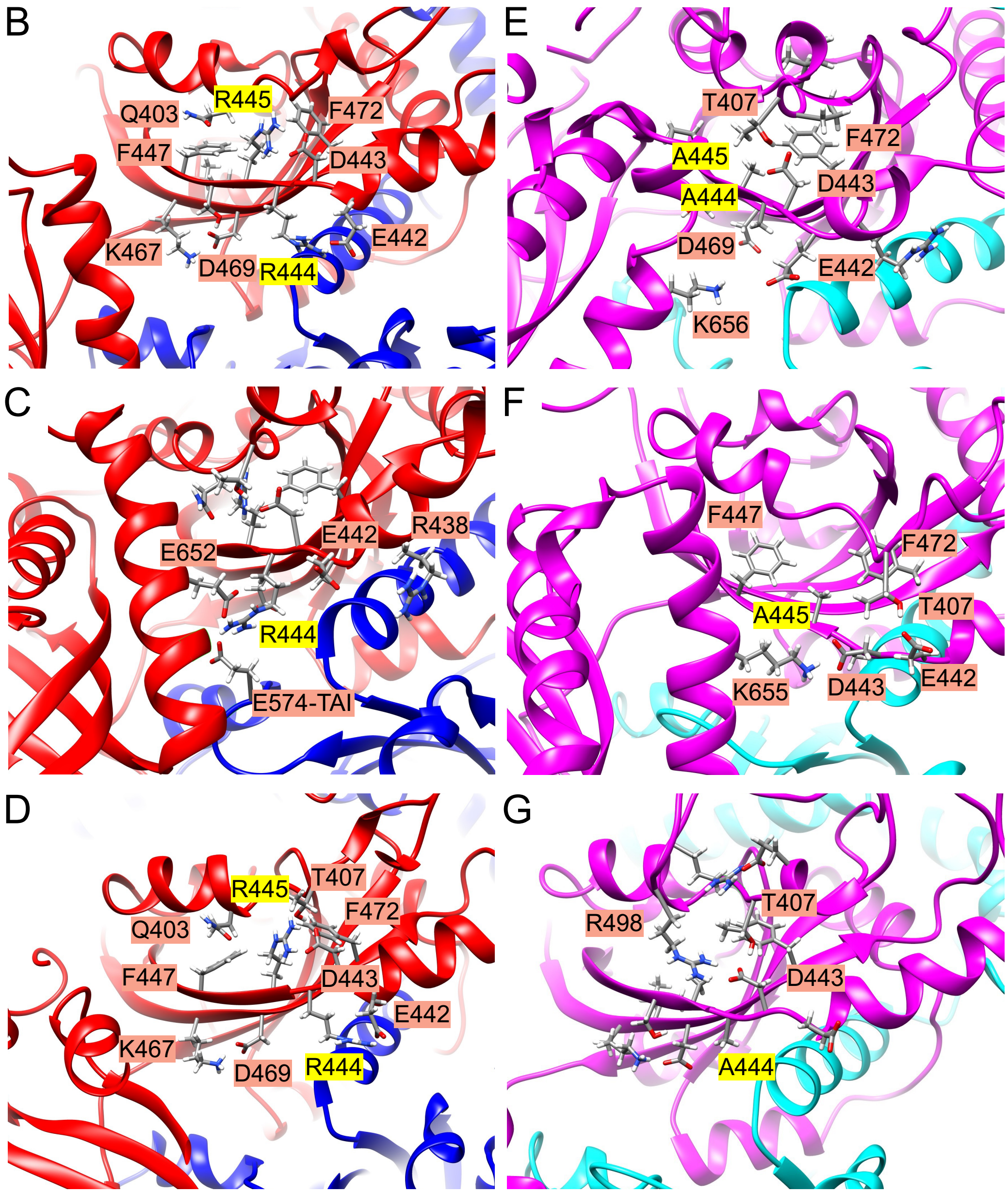

Fig. S11 (*continued*). (*B-G*) Detail views of the wild-type interaction cluster within and between domains (*B-D*) and of the mutated GCE site and its vicinity (*E-G*). Three representative configurations from five repeated MD trajectories are shown. Interacting (orange labels) and mutated (yellow labels) residues are indicated. Note that individual panels are shown in different views. As expected from the relatively short duration of MD simulation, most structural changes resulting from the mutation manifested themselves in the vicinity of the mutation. As in the previous MD, wild-type configurations (*B-D*) were stabilized by a local network of electrostatic interactions (salt bridges between R445 and D443, and R444 and E442 or, alternatively, between R444 and E574 in the adjacent TAI). Consequently, the subunit interfaces were not affected. In contrast, the double-mutant (*E-G*) adopted a greater variety of configurations due to the disruption of the local electrostatic network. As a result, the hydrophobic core of the PAS-A β-barrel (F447, F472) was exposed and the negative charge of E442 and D443 was balanced by heterologous interactions with positively charged residues, either from the adjacent PAS-B domain (K655 and K656) or from a flexible loop of PAS-A (R498).

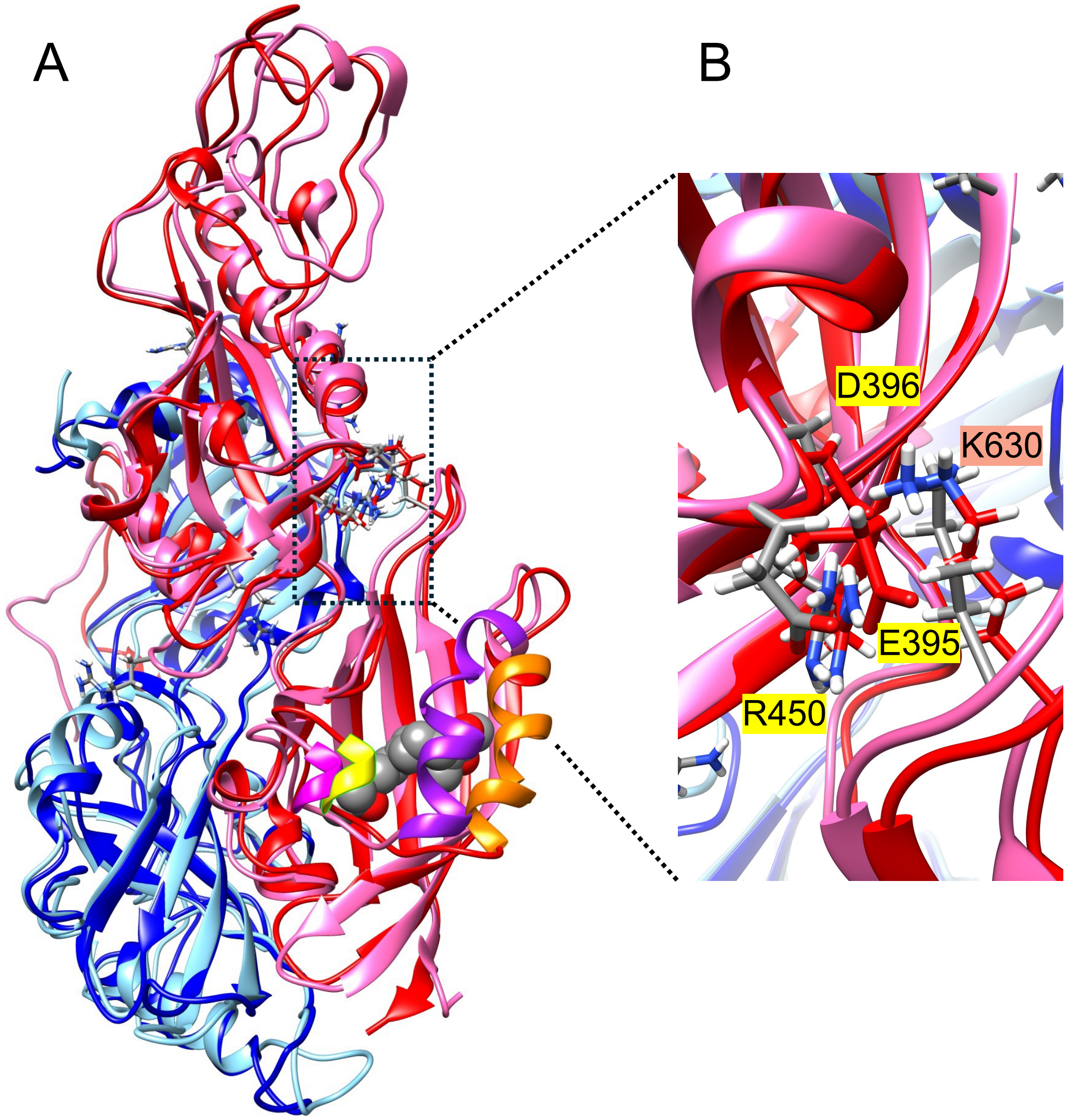

Fig. S12. Effects of JH III binding on the structure of PAS-A and PAS-B domains. (*A*) Comparison of a ribbon diagram of ligand-free heterodimer (GCE, pink; TAI, cyan; AlphaFold 3 structure relaxed by MD simulation as in Fig. 6, main text) with that of JH III (spacefill in CPK colors) bound heterodimer (GCE, red; TAI, blue; structure relaxed by further 300 ns of MD simulation). GCE PAS-A residues that were subjected to mutagenesis are shown in stick representation. GCE PAS-B helices Eα and Fα that undergo ligand-induced rearrangements are highlighted in magenta (ligand-free) and yellow (liganded), and purple (ligand-free) and orange (liganded), respectively. The box indicates interacting charged residues at the GCE PAS-A/B domain interface as shown in detail in (*B*), where residues subjected to mutagenesis are indicated by yellow labels.

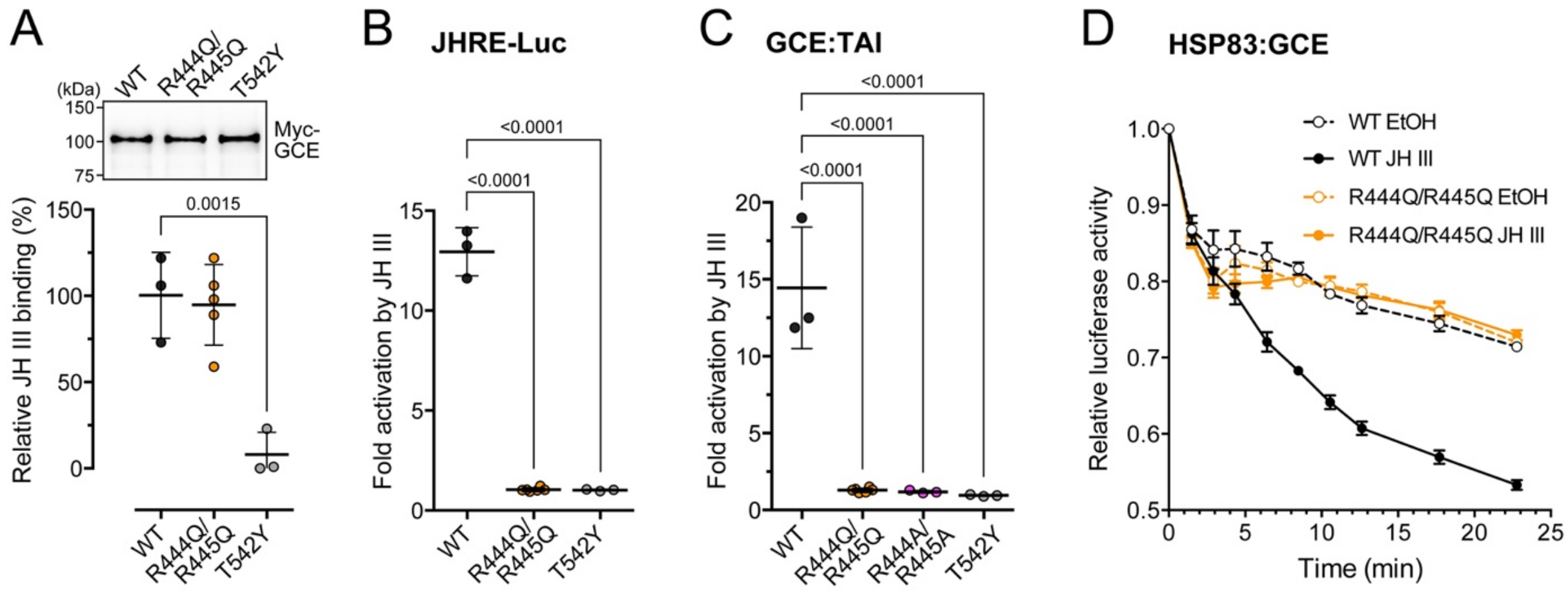

Fig. S13. Impact of mutating the conserved R444 and R445 residues to uncharged glutamine. (*A*) Binding of [^{3}H]-JH III by the *in-vitro* translated GCE$^{R444Q/R445Q}$ protein was unaffected to the control (WT) GCE (100%); GCE^{T542Y} is a JH nonbinding mutant. Immunoblot (*top*) shows equal expression of all variants. (*B*) GCE$^{R444Q/R445Q}$ was unable to induce the *JHRE-luc* reporter in response to JH III (100 nM) in the Kc167 cells. Data are plotted relative to the luciferase activity with vehicle alone. (*C*) Substitution of R444 and R445 with glutamine or alanine prevented dimerization with TAI in the two-hybrid assay. Activation by JH III (100 nM) is plotted as fold change relative to the luciferase activity measured for each GCE variant in the presence of vehicle alone, a value arbitrarily set to 1. Data in (*A*-*C*) appear as mean ± SD from indicated data points; *P* values (one-way ANOVA) are shown only for significant differences. (*D*) JH-induced dissociation of GCE from HSP83 was impaired by the R444Q/R445Q mutation relative to GCE^{WT} as determined in the NanoBiT assay in the presence or absence of 1 μM JH III. The traces are shown as average of six measurements with the error bars representing SEM.

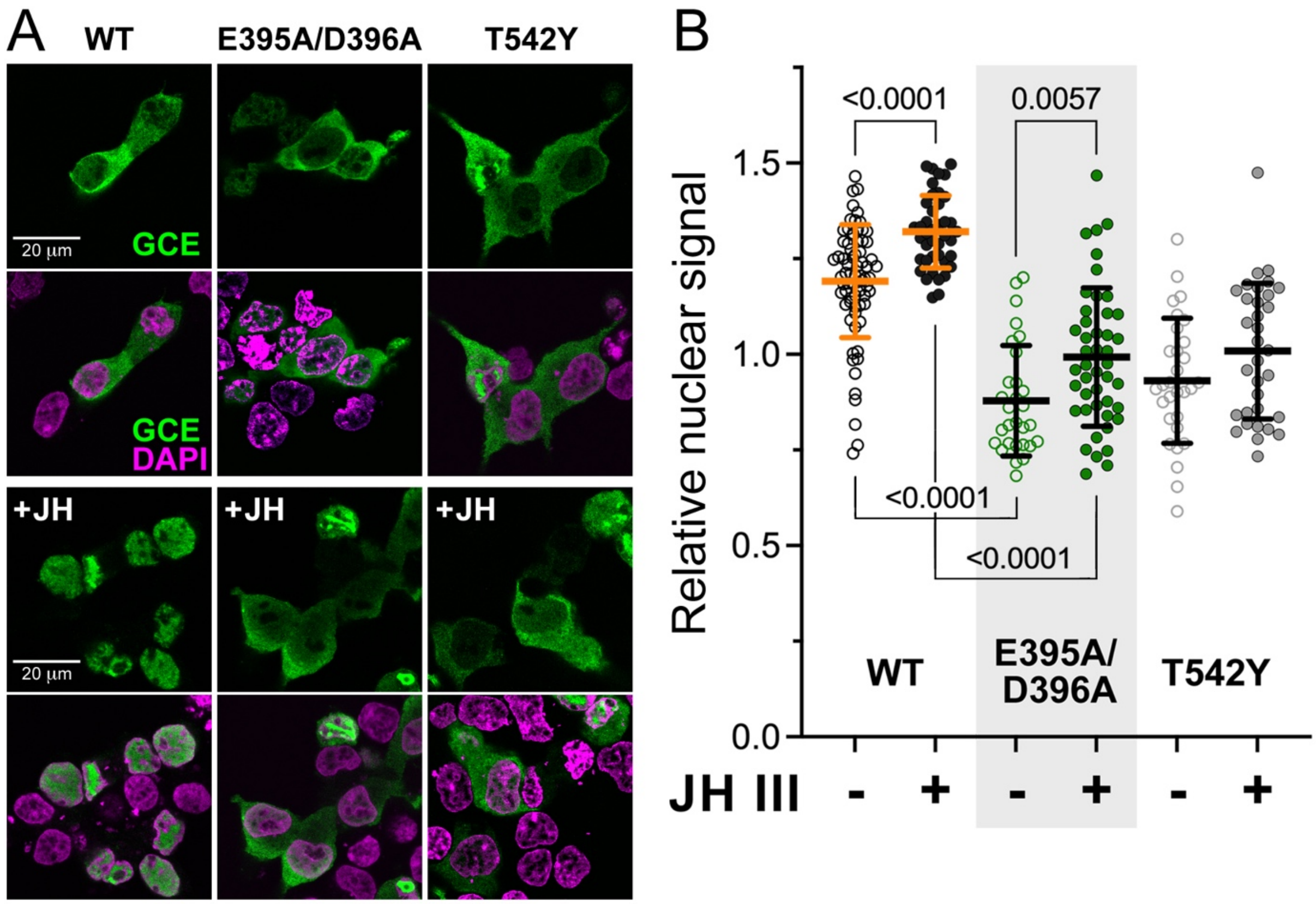

Fig. S14. (*A*) GCE WT and indicated mutant variants carrying an N-terminal Myc-tag were transiently expressed in HEK293 cells. After 1-hour incubation with 1 µM JH III (+ JH) or ethanol, cells were stained with anti-Myc antibody (green) and DAPI (magenta). The images are single confocal slices; the scale bar (20 µm) applies to all panels. (*B*) Cellular localization of GCE was assessed as integrated pixel densities in confocal slices from stained transfected cells; each dot represents the nuclear-to-total signal ratio for an individual cell, either from the vehicle control or treated by JH III. While the $GCE^{E395A/D396A}$ mutant protein responded to JH III by translocating to the nuclei, relative to the WT control it showed higher cytoplasmic retention similar to that of the GCE^{T542Y} variant both in the presence and absence of JH III. The nuclear enrichment of GCE^{T542Y} induced by JH III was not statistically significant.

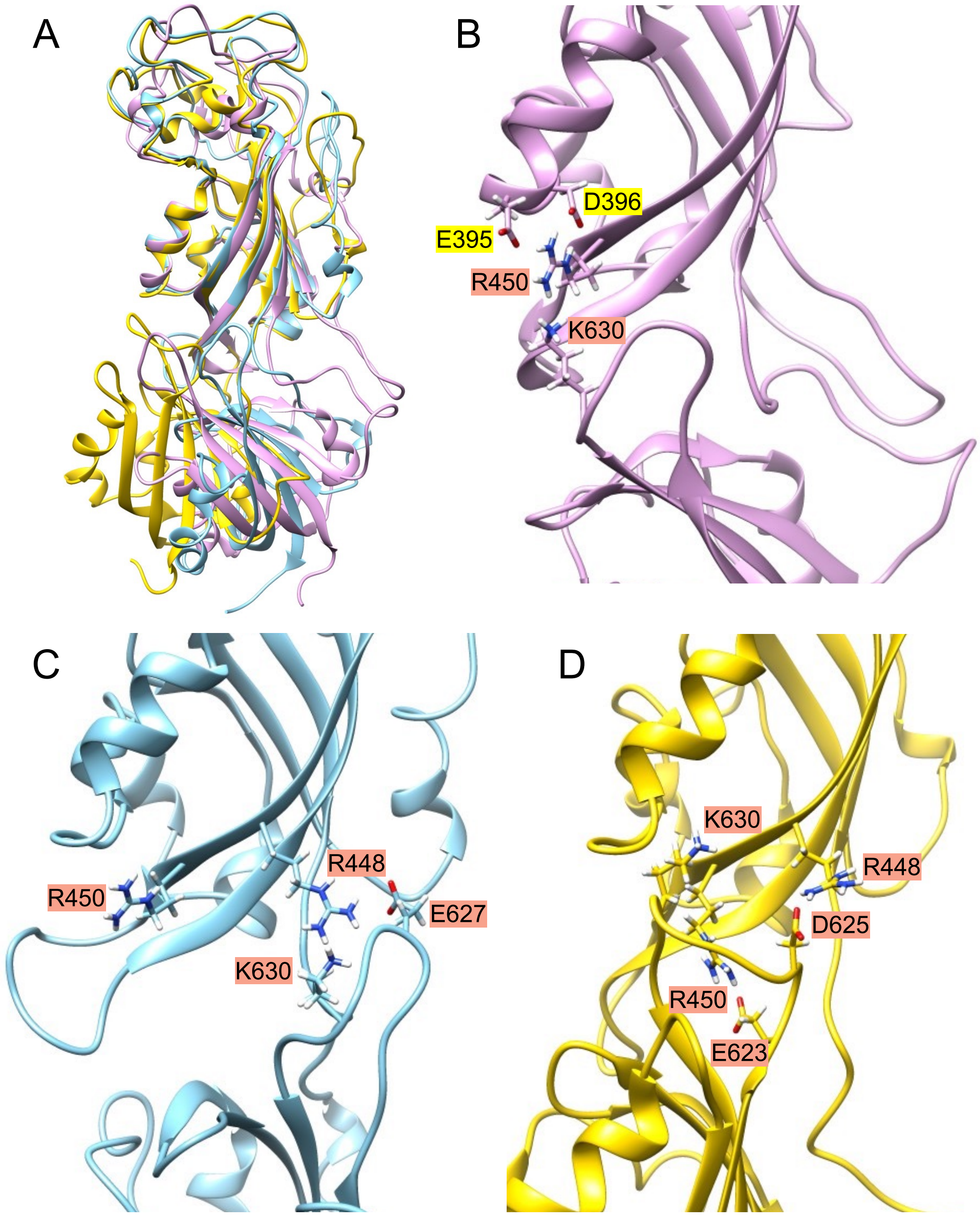

Fig. S15. Structural consequences of the GCE E395A/D396A double mutation. MD simulation of GCE PAS-A/B domain model bearing the double mutation. Two 3-μs all-atom MD simulations with different outcomes (cyan and yellow) compared with wild-type GCE (pink) and RMSD aligned with respect to the PAS-A domain (*A*). (*B-D*) Detailed views (enlarged, cropped and rotated relative to that shown in panel *A*) of the wild-type (*B*, pink) and the two conformations (*C*, cyan and *D*, yellow) resulting from the E395A/D396A mutation. Mutated residues (yellow labels) and interacting residues (orange labels) at the interface are shown as stick representation. Note the altered position of R450.

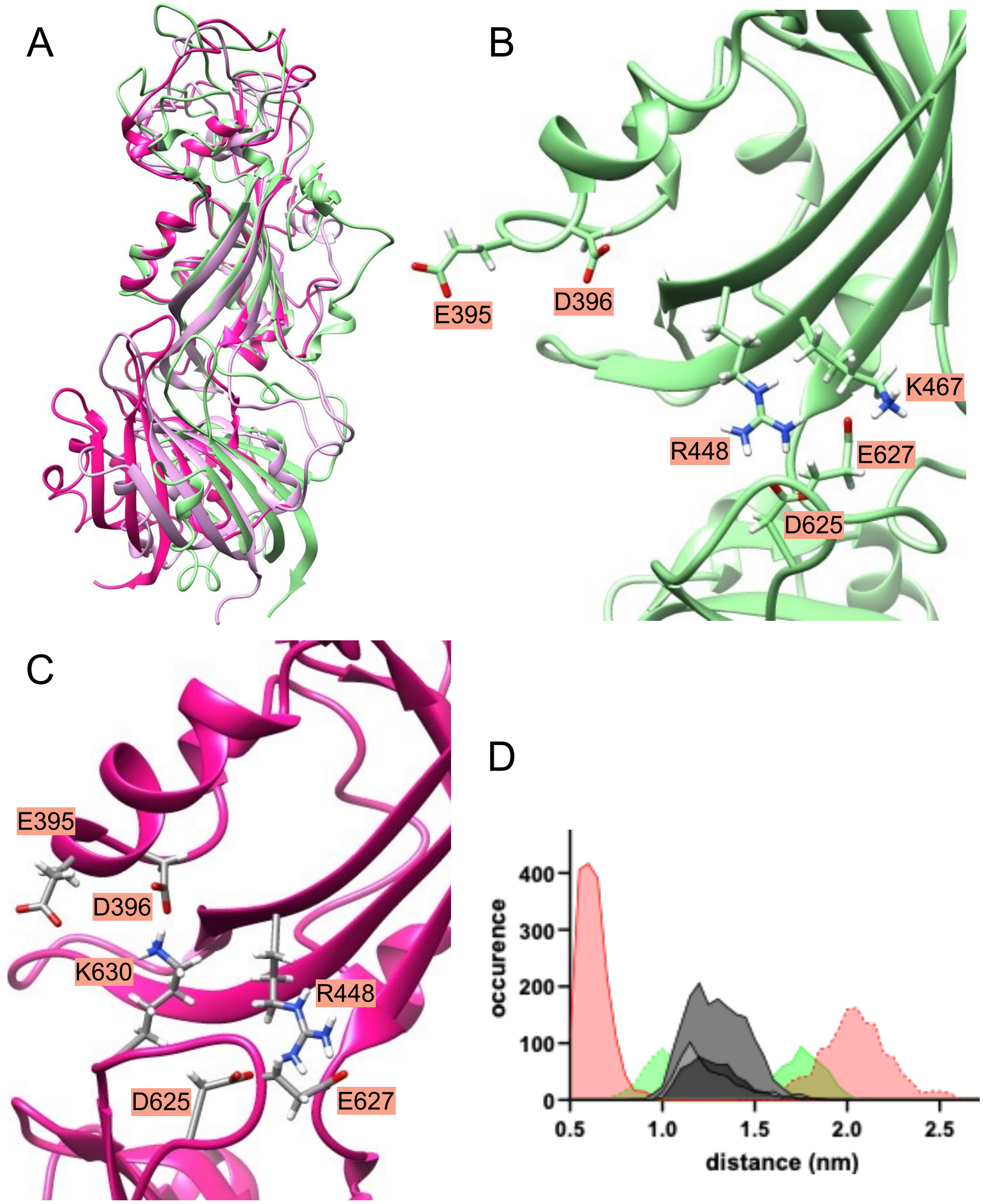

Fig. S16. Structural consequences of R450A mutation. MD simulation of GCE PAS-A/B domain model bearing the mutation. Two 3-µs all-atom MD simulations with different outcomes (green and magenta) compared with wild-type GCE (pink) and RMSD aligned with respect to the PAS-A domain (*A*). (*B,C*) Detailed views, identical to that in Fig. S15 and enlarged and cropped with respect to that shown in (*A*). Residues at the interface are shown as stick representation. Note the position of E395. (*D*) Distributions of distance between PAS-A E395 (or A if mutated) and K630 C atoms in wild-type GCE (shades of grey for repeats) and the variants R450A (green) and E395A/D396A (red).

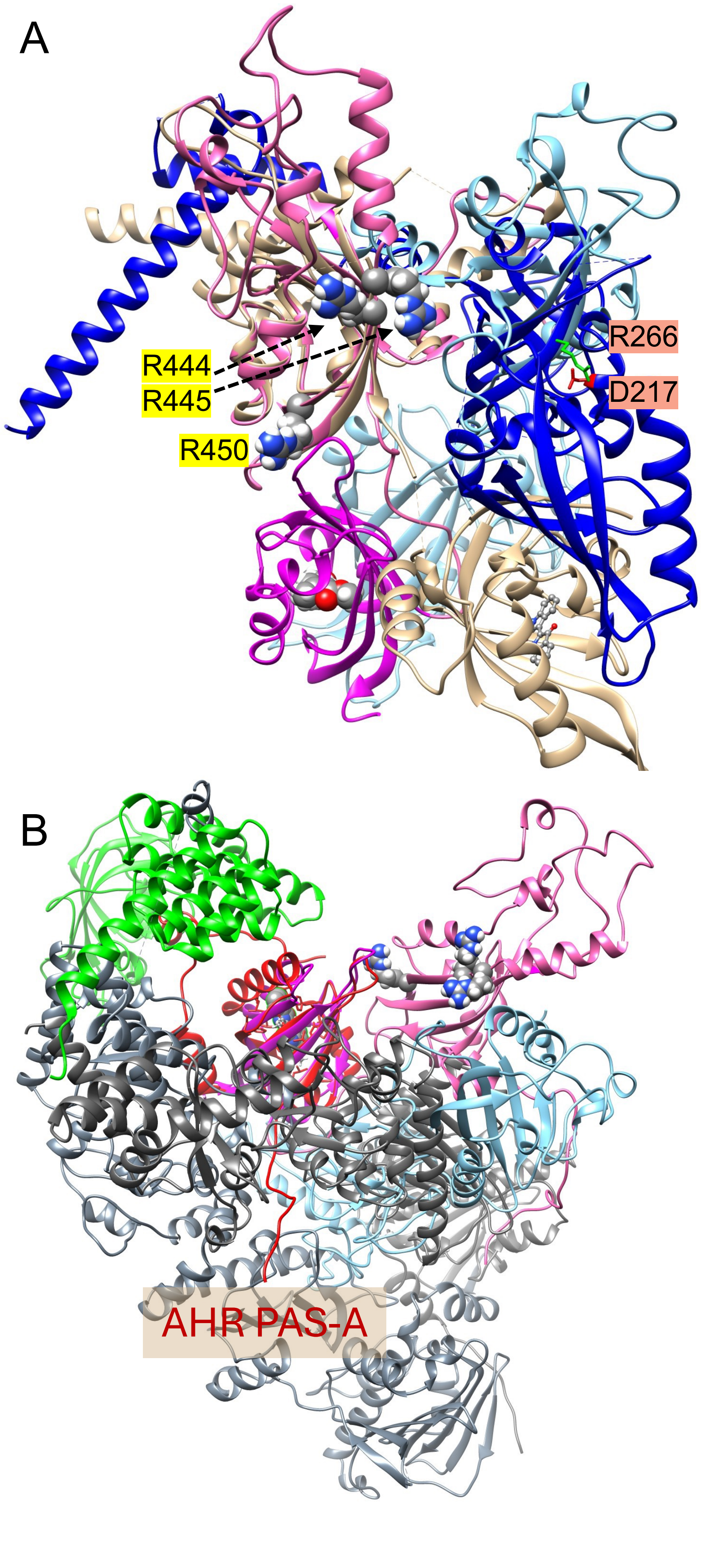

Fig. S17. Comparison of PAS domain disposition in GCE:TAI and AHR:ARNT dimers.
(*A*) Comparison of MD relaxed GCE:TAI model (ribbon representation; PAS-A, pink; PAS-B, magenta; TAI, cyan; JH III shown in spacefill and CPK colors) with AHR:ARNT PAS-A structure [PDB 8XSB (4); AHR, tan; ARNT, blue; indirubin shown in ball-and-stick and CPK colors]. Structures were aligned using Cα RMSD of PAS-A domains. Mutated GCE PAS-A residues are shown in spacefill and highlighted by yellow labels. The conserved ARNT R266 (green sticks) and its interacting partner D217 (red sticks) are indicated by orange labels.
(*B*) Comparison of MD relaxed GCE:TAI model (ribbon representation; PAS-A, pink; PAS-B, magenta; TAI, cyan; JH III shown in ball-and-stick and CPK colors) with AHR PAS-B [PDB 7ZUB (5), red ribbons] in complex with indirubin (spacefill, CPK colors), HSP90 dimer (gray ribbons) and XAP2 (green ribbon). Structures were aligned using Cα RMSD between PAS-B domains. Mutated GCE PAS-A residues shown in spacefill. Putative position of AHR PASA domain is indicated.

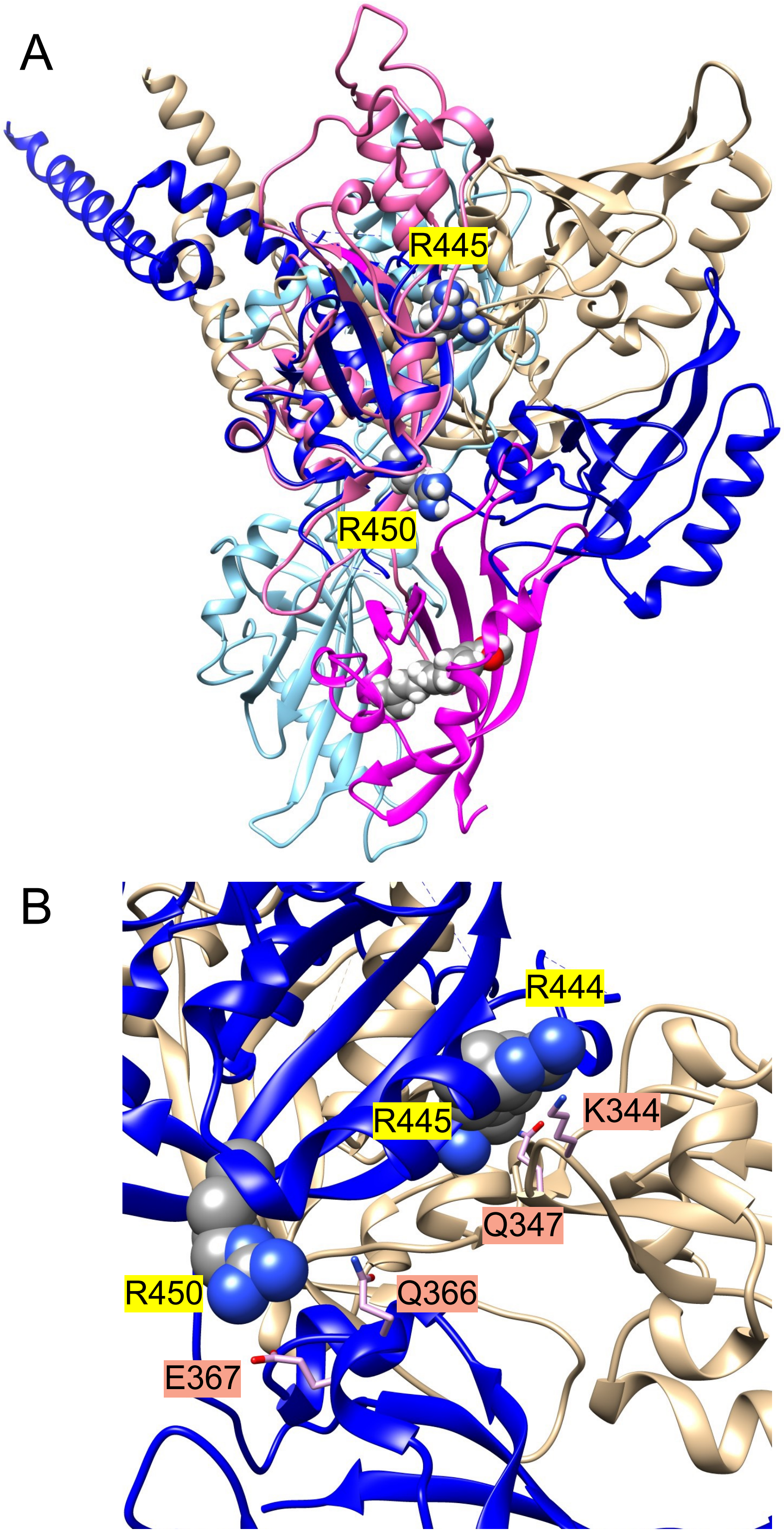

Fig. S18. Comparison of PAS domain disposition in GCE:TAI and CLOCK:BMAL1 dimers. (*A*) Comparison of MD relaxed GCE:TAI model (ribbon representation; PAS-A, pink; PAS-B magenta; TAI, cyan; JH III shown in spacefill and CPK colors) with CLOCK:BMAL1 structure [PDB 4F3L (6); CLOCK, tan; BMAL1, blue]. Structures were aligned using Cα RMSD of PAS-A. Mutated GCE PAS-A residues are shown in spacefill with yellow labels.
(*B*) Details of the interacting interfaces around the arginine residues in BMAL1 (blue) that are conserved with R444, R445 and R450 in GCE (spacefill and yellow labels; the numbering corresponds to GCE sequence; see Fig. S1). Interacting residues in CLOCK (tan) are shown in stick representation with orange labels.

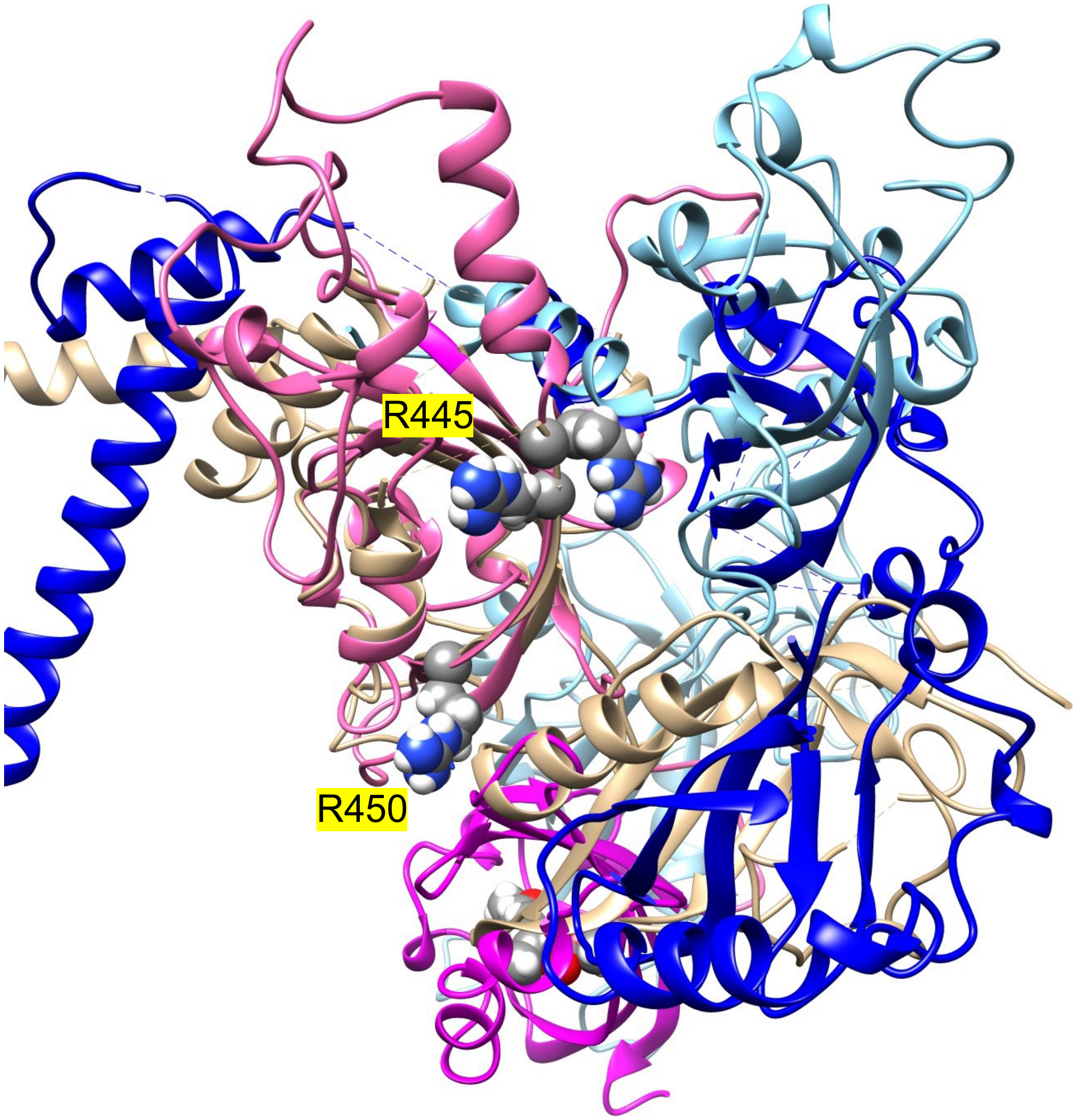

Fig. S19. Comparison of PAS domain disposition in GCE:TAI and HIF-1α:ARNT dimers. (*A*) Comparison of MD relaxed GCE:TAI model (ribbon representation; PAS-A, pink; PAS-B magenta; TAI, cyan; JH III shown in spacefill and CPK colors) with AHR:ARNT structure [PDB 4ZPR (1), HIF-1α, tan; ARNT, blue). Structures were aligned using PAS-A Cα RMSD and DNA was omitted for clarity. Mutated GCE PAS-A residues are shown in spacefill.

Supplementary Discussion

Structural implications of the mutations for related complexes

Given the conservation of GCE R445 and R450 in equivalent positions of many PAS-A domain sequences, we compared our model with available high-resolution structures. Since the initial GCE:TAI model was based on a training set encompassing also the structures used for comparison, one must consider that any structural similarity, *e.g.*, domain disposition, could result from this initial model bias. Nevertheless, the comparisons provide novel clues as to the possible roles of the conserved residues across PAS domain heterodimers and allow us to speculate about mechanistic implications.

The most relevant and ligand activated complex is the AHR:ARNT dimer. Recently, AHR:ARNT structure encompassing both PAS-A and PAS-B and bound to DNA was published (4). Comparison of this crystal structure (liganded with indirubin, PDB 8XSB) with our core complex, MD relaxed AlphaFold 3 model (Fig. 6, main text) with docked JH III (Fig. S17*A*) indicates that the disposition of the PAS-A domains of AHR and ARNT is similar to that of the GCE:TAI complex, suggesting that R445 may play a similar role in stabilizing the domain interface within the heterodimer. Interestingly, although AHR PAS-B only interacts with ARNT PAS-B, the interface may resemble that predicted for GCE:TAI. Note that the conserved R450 equivalent R266 in ARNT interacts with D217 of the same PAS-A domain within this complex (Fig. S17*A*).

As mentioned in our previous analysis (3) the structure of AHR PAS-B within the chaperone complex is incompatible with the predicted PAS domain disposition of GCE, and HSP83 might also interfere with TAI binding (Fig. S17*B*). If we assume that AHR and GCE PAS-B domains interact with HSP90/83 in a similar way, then heterodimer formation would require significant rearrangement of the bound PAS-B domain or its partial release. This might be facilitated by formation of the PAS-A/B domain interface (encompassing R450 in GCE) which would then stabilize the PAS-A domain in a position that sterically interferes with rebinding to HSP90/83. R445 at the PAS-A interface between GCE and TAI will similarly stabilize the heterodimer, which is likely to be sterically incompatible with HSP90/83 binding (see the overlap of HSP90 and TAI, Fig. S17*B*).

In the context of CLOCK:BMAL1, it is the partner BMAL1 which exhibits the conserved arginine residues (Fig. S1) and the PAS-A structure lines up well with the corresponding domain in the GCE model (Fig. S18*A*). The domain disposition and interaction pattern within this structure is different from the GCE:TAI model but the conserved arginines are part of the interdomain interfaces. Unlike GCE, the R444/5 cluster equivalent in BMAL1 (R244/5 according to the PDB 4F3L numbering) interfaces with the PAS-B of CLOCK (Fig. S18*B*). While the BMAL1 R250 equivalent of R450 in GCE contacts its own PAS-B domain, the contact point is with residues Q366 and E367 in helix Dα (Fig. S18*B*).

The conserved arginines map onto similar interfaces in HIF-1α:ARNT (Fig. S19). R165 (GCE R445 equivalent) is part of HIF-1α PAS-A and ARNT PAS-A interface, which interacts with helix Aα and possibly with the linker between the PAS-A and PAS-B domains of ARNT (not resolved in the structure). R170 (GCE R450 equivalent) maps to HIF-1α PASA-PASB interface, similar as in GCE. However, unlike in GCE, it maps to the vicinity of helix Fα. This reflects the different disposition of PAS-B which in HIF-1α is displaced and rotated compared to that in GCE.

In summary, the conserved arginines, which exhibit the most severe loss of function upon mutation, map onto previously proposed domain and subunit interfaces in other PAS heterodimers. However, the pattern of their interaction reflects different disposition of the PAS domains relative to one another.

Table S1. Primers used for *D. melanogaster gce* mutagenesis

Mutant	Forward primer (5'→ 3')*
E395A/D396A	CATGCAGATTACCCACCCAGCGGCTCAGAACATGCTGAAGCAGC
E433A	GCCAGCGCAGCAAGGCCGAGGCGGACGCCATTGATCGC
D437A	GGCCGAGGAGGACGCCATTGCTCGCAAGCTGCGCGAGG
R444A	CGCAAGCTGCGCGAGGATGCCCGCTCCTTTCGCGTGCGCC
R445A	CAAGCTGCGCGAGGATCGCGCCTCCTTTCGCGTGCGCC
R444A/R445A	CAAGCTGCGCGAGGATGCCGCCTCCTTTCGCGTGCGCC
R444Q/R445Q	CGCAAGCTGCGCGAGGATCAACAATCCTTTCGCGTGCGCCTG
R450A	CGCCGCTCCTTTCGCGTGGCCCTGGCCCGCGCCGGACCGC
R453A	CCTTTCGCGTGCGCCTGGCCGCCGCCGGACCGCGCTCCGAGC
R473A	GTGAAGATCGACGGATGCTTCGCCCGCAGTGATGAGGCCC
R474A	GATCGACGGATGCTTCCGCGCCAGTGATGAGGCCCCGCG
D476A	GATGCTTCCGCCGCAGTGCTGAGGCCCCGCGCGGCG
R480A	CGCAGTGATGAGGCCCCGGCCGGCGTGCGCTCCAACC
T542Y	CCAATACCCTGGAGTACAAGTACCGCCACCTGATCGATGG

*The mutated nucleotides are underlined. Reverse primers are exact reverse complementary to the forward primers.

Table S2. Primers used for RNAi knockdown in *D. melanogaster* Kc167 cells

dsRNA/gene	Forward (F) and reverse (R) primers (5'→ 3')*
egfp-(F)	taatacgactcactatagggATGGTGAGCAAG
egfp-(R)	taatacgactcactatagggCTTGTACAGCTC
gce-(F)	taatacgactcactatagggTTGCATTGCTCCAGGACC
gce-(R)	taatacgactcactatagggGCCTGGGATCGTGATGC
Met-(F)	taatacgactcactatagggCATTGACCACACTCACCGAC
Met-(R)	taatacgactcactatagggCAACAGTGACTACGGCGAGA
tai-(F)	taatacgactcactatagggGCTTGTAGTCTGCGTGCTGT
tai-(R)	taatacgactcactatagggGTCACCCACACGACCTATCC
Hsp83-(F)	taatacgactcactatagggGGCACGGTTGTTGCTGTC
Hsp83-(R)	taatacgactcactatagggTGAGGAGCTGAACAAGACC

*Each gene-specific primer sequence is preceded by the T7 RNA polymerase promoter sequence (lowercase) included for dsRNA synthesis.

References

1. D. Wu, N. Potluri, J. Lu, Y. Kim, F. Rastinejad, Structural integration in hypoxia-inducible factors. *Nature* **524**, 303–308 (2015).
2. F. Madeira *et al*., The EMBL-EBI Job Dispatcher sequence analysis tools framework in 2024. *Nucleic Acids Res*. **52(W1)**, W521–W525 (2024).
3. D. Sedlak *et al*., Unique and common agonists activate the insect juvenile hormone receptor and the human AHR. *J. Mol. Biol.* **437**, 168883 (2025).
4. X. Diao *et al*., Structural basis for the ligand-dependent activation of heterodimeric AHR-ARNT complex. *Nat. Commun*. **16**, 1282 (2025).
5. J. Gruszczyk *et al*., Cryo-EM structure of the agonist-bound Hsp90-XAP2-AHR cytosolic complex. *Nat. Commun*. **13**, 7010 (2022).
6. N. Huang *et al*., Crystal structure of the heterodimeric CLOCK:BMAL1 transcriptional activator complex. *Science* **337**, 189–194 (2012).
